# Single-cell and spatial landscape reveals impaired tertiary lymphoid structure maturation in *Helicobacter pylori*–associated gastric cancer

**DOI:** 10.3389/fimmu.2026.1908868

**Published:** 2026-08-13

**Authors:** Nenglin Zhang, Qiuyu Jiang, Qian Ma, Haisheng Huang, Lijie Ma, Shuping Zhou

**Affiliations:** 1 Department of Gastroenterology, The First Affiliated Hospital of Anhui University of Science and Technology, Huainan, China; 2 Department of Gastroenterology and Hepatology, Shanghai Institute of Liver Diseases, Zhongshan Hospital of Fudan University, Shanghai, China; 3 Department of Dermatology, The Children’s Hospital of Fudan University, Shanghai, China; 4 Department of General Surgery, Zhongshan Hospital, Fudan University, Shanghai, China

**Keywords:** gastric cancer, *Helicobacter pylori*, HSP70, immunotherapy, tertiary lymphoid structure

## Abstract

**Background:**

Tertiary lymphoid structures (TLSs) are key immune niches involved in antitumor immunity. However, their functional impact depends on spatial localization, maturation status, and cellular composition within the tumor immune microenvironment (TIME). Despite the recognized role of *Helicobacter pylori* (*H. pylori*) in gastric carcinogenesis and immune modulation, its influence on TLS architecture and function in gastric cancer remains poorly understood.

**Methods:**

We integrated public single-cell RNA sequencing (scRNA-seq; HRA000704, GSE150290, and GSE249874; n=36), spatial transcriptomics (ST; GSE251950; n=9), and multiplex immunofluorescence (mIF) staining (n=90) to comprehensively profile TLSs across *H. pylori* strata. Subsequently, Kaplan–Meier (KM) analysis, restricted cubic spline (RCS) analysis, receiver operating characteristic (ROC) analysis, and concordance index (C-index) assessment were employed to determine the predictive value of distinct TLS-related features on clinical outcomes.

**Results:**

*H. pylori*-positive GC showed preferential peritumoral TLS localization and reduced TLS maturation. Within TLSs, *H. pylori*-positive tumors showed accumulation of HSP70^+^ B cells and reduced representation of ZNF683^+^ CD8^+^ T cells. This compositional shift was associated with disrupted CD21^+^ follicular dendritic cell (FDC) networks and CD23^+^ germinal center-like structures. Based on these reciprocal intra-TLS immune patterns, two TLS compositional states were defined. TLS type 1, marked by HSP70^+^ B-cell enrichment and ZNF683^+^ CD8^+^ T-cell reduction, was enriched in *H. pylori*-positive tumors, whereas TLS type 2 showed the opposite pattern and was associated with favorable overall survival (OS). Incorporating TLS composition into the conventional clinicopathological model increased the 5-year area under the ROC curve (AUC) from 0.847 to 0.910.

**Conclusions:**

Our study identifies an *H. pylori*-associated TLS compositional state characterized by HSP70^+^ B-cell enrichment and reduced ZNF683^+^CD8^+^ T-cell representation. Incorporating TLS composition into clinicopathological models provided incremental prognostic information in gastric cancer.

## Graphical Abstract

**Figure:**
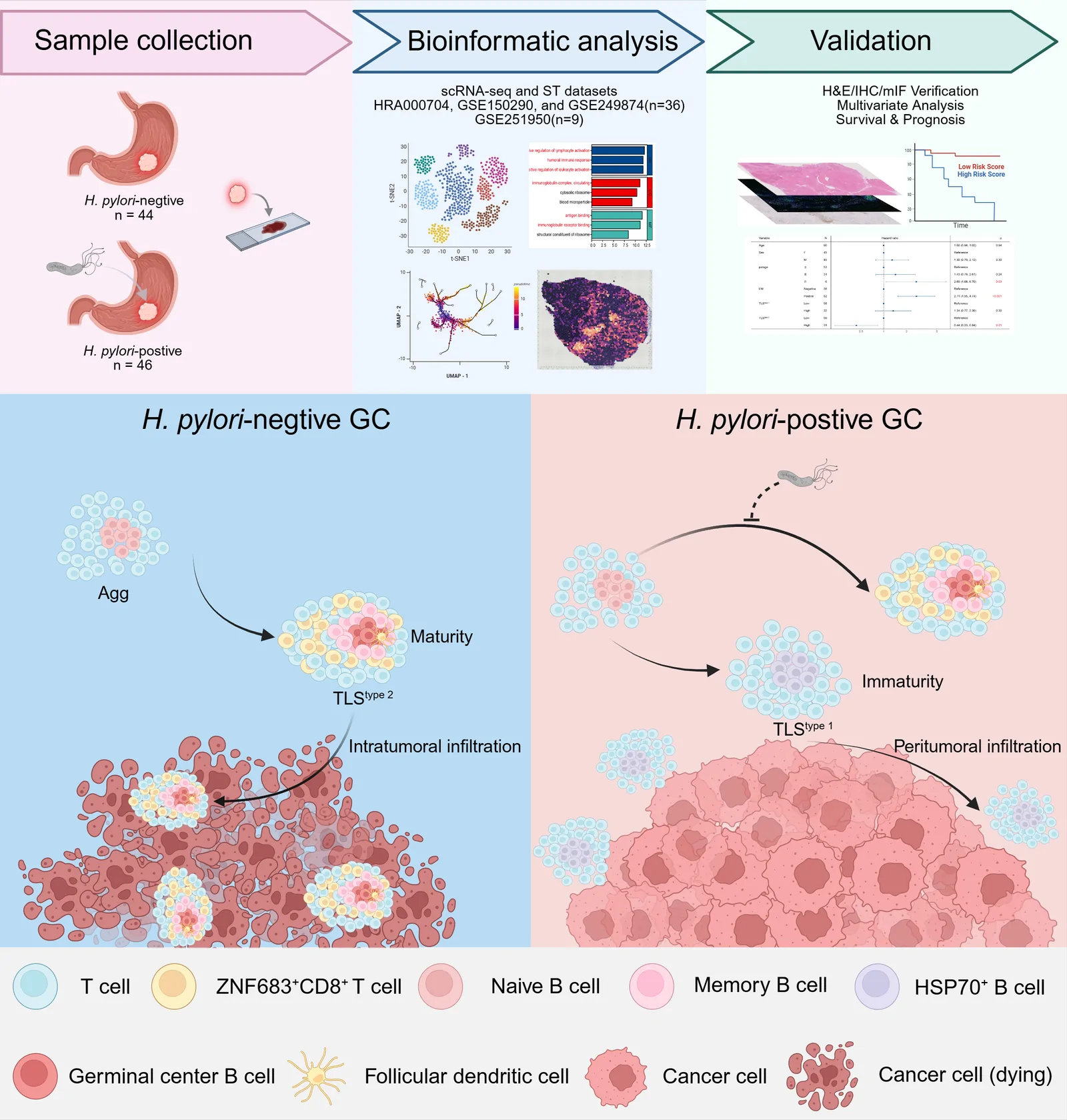
The study integrates scRNA-seq, ST and mIF analyses to characterize TLSs in *H. pylori*-negative and *H. pylori*-positive GC. *H. pylori*-negative tumors are characterized predominantly by mature, intratumoral TLSs with increased ZNF683^+^CD8^+^ T-cell representation, whereas *H. pylori*-positive tumors show predominantly immature, peritumoral TLSs enriched in HSP70^+^ B cells.

## Highlights

- *H. pylori*-positive GC exhibits peritumoral TLS localization and impaired TLS maturation.
- *H. pylori*-positive tumors show B/plasma-cell enrichment and reduced T/NK-cell representation.
- HSP70^+^ B-cell enrichment and ZNF683^+^CD8^+^ T-cell depletion define a less mature TLS compositional state in *H. pylori*-positive tumors.
- Incorporating TLS composition into clinicopathological models provides incremental prognostic value in GC.

## Introduction

Gastric cancer (GC) constitutes a substantial global disease burden, accounting for the fifth highest incidence and mortality rates among all malignancies worldwide (1). *Helicobacter pylori* (*H. pylori*), a Gram-negative pathogen that chronically colonizes the human gastric mucosa, constitutes the principal infectious risk factor for GC development (2). Designated a Group 1 carcinogen by the World Health Organization (WHO), chronic *H. pylori* infection underlies at least 75% of GC cases worldwide (3). Emerging research has illuminated the capacity of *H. pylori* to sculpt the tumor immune microenvironment (TIME), with mechanistic studies revealing that persistent infection drives chronic mucosal inflammation and fosters an immunosuppressive niche that facilitates neoplastic progression (4–8). Meanwhile, *H. pylori*-positive patients with advanced disease exhibited substantially elevated non-response rates to anti-PD-1 therapy (9). Among those metastatic GC patients receiving immune checkpoint inhibitor (ICI), *H. pylori*-positive patients presented significantly inferior progression-free survival (PFS) and overall survival (OS) (10). Murine model studies confirmed that *H. pylori* directly attenuated immunotherapeutic response (11). However, the mechanisms of *H. pylori*-mediated immune tolerance in GC remained incompletely understood.

Tertiary lymphoid structures (TLSs) are ectopic lymphoid aggregates that develop in non-lymphoid tissues under pathological conditions such as chronic inflammation, infection, and cancer. They are typically composed of B cells, T cells, follicular dendritic cells, and associated stromal and vascular components (12, 13). Studies in GC have shown that the presence of TLSs, high TLS levels, and TLS-associated B-cell infiltration are generally associated with stronger antitumor immunity and prolonged patient survival (14, 15). However, the clinical significance of TLSs is not determined solely by their abundance. For example, the maximum TLS diameter was associated with patient survival, whereas TLS density did not consistently demonstrate independent prognostic value (16). More recent studies have shown that mature TLSs containing germinal centers and TLSs located at the invasive margin are associated with more favorable clinical outcomes (17). TLSs in primary tumors and peritoneal metastases also differ substantially in their maturation status and immune context (18). Single-cell and spatial transcriptomic analyses have further revealed the spatial distribution of TLSs in GC and their potential clinical significance (19). Collectively, these findings indicate that TLS abundance alone does not fully capture the biological and clinical heterogeneity of TLSs in GC, which may also depend on their spatial location, maturation status, and internal cellular composition.

Previous studies have shown that chronic *H. pylori* infection induces organized lymphoid follicles in non-neoplastic gastric mucosa, partly through the CXCL13–CXCR5 axis, and that these structures regress following successful eradication therapy (20–22). However, whether this infection-associated lymphoid organization persists or is reprogrammed within GC remains unknown. Specifically, it is unclear whether *H. pylori* infection is associated with changes in the cellular composition, spatial organization, and maturation status of TLSs in GC and whether these changes have clinical relevance.

In this study, we integrated single-cell RNA sequencing (scRNA-seq), spatial transcriptomics (ST), and multiplex immunofluorescence (mIF) to investigate TLS heterogeneity in GC according to *H. pylori* status. scRNA-seq was used to identify infection-associated immune-cell states, ST to assess their spatial distribution in TLS-enriched regions, and mIF to validate their intra-TLS localization and relationships with follicular architecture and maturation markers. We further evaluated the association between TLS cellular composition and clinical outcomes.

## Materials and methods

### Human tissue samples

We enrolled 90 GC patients to evaluate associations between *H. pylori* status and TLS characteristics (**Supplementary Tables 1, 2**). Infection status was determined by ¹³C-urea breath test and immunohistochemistry (IHC). The inclusion criteria were as follows: (1) curative surgery for GC at the First Affiliated Hospital of Anhui University of Science and Technology between December 2016 and December 2023; (2) no prior history of neoadjuvant or preoperative therapy; (3) availability of complete follow-up data for overall survival (OS) ascertainment. Patients were excluded if they presented with inadequate pathological material (fewer than 5 slides) or were lost to follow-up.

### Hematoxylin and eosin and IHC staining and TLS evaluation

Formalin-fixed, paraffin-embedded (FFPE) GC tumor tissues were sectioned at 4μm. For H&E staining, sections were deparaffinized, rehydrated, and stained with hematoxylin for 5 minutes and eosin for 2 minutes. For IHC, rehydrated sections underwent microwave antigen retrieval in EDTA (pH 9.0) or citrate buffer (pH 6.0) for 20 minutes. Endogenous peroxidase and non-specific binding were blocked using 3% H_2_O_2_ (10 minutes) and 5% normal goat serum (30 minutes), respectively. Sections were incubated overnight at 4 °C with primary antibodies against CD20 (Abcam, ab9475, 1:200), CD21 (Abcam, ab75985, 1:8000), CD23 (Abcam, ab92495, 1:200) and H. pylori (Abcam, ab172611, 1:13000) followed by HRP-conjugated secondary antibody incubation for 30 minutes, DAB visualization, and hematoxylin counterstaining. Finally, TLSs were independently quantified by two blinded pathologists based on H&E and IHC markers (CD20 for B-cell zones, CD21 for follicular dendritic cell (FDC) networks and CD23 for germinal center-like structures). TLSs were subsequently classified into two spatial subtypes based on their anatomical location: intra-tumoral (iTLS, within the tumor boundary) or peri-tumoral (pTLS, outside the tumor boundary) (19).

### Data collection

Public scRNA-seq datasets HRA000704 (23), GSE150290 (24), and GSE249874 (25) included 36 GC patients (25 *H. pylori*-positive and 11 *H. pylori*-negative) after excluding non-GC tissues. Additional datasets included spatial transcriptomic data from nine primary GC tissues (GSE251950) (26), an *H. pylori*-annotated microarray cohort for TIDE analysis (GSE13195) (27), TCGA-STAD bulk RNA-seq data for signature and survival analyses (28), and the pembrolizumab-treated PRJEB25780 cohort for immunotherapy-response validation (29).

### Quality control and cell annotation of scRNA-seq data

Public scRNA-seq datasets HRA000704, GSE150290, and GSE249874 were included in this study (**Supplementary Table 3**). All included samples were generated using the 10x Genomics single-cell RNA-seq platform. To ensure sample type consistency across datasets, only GC tumor samples were retained for the present analysis, whereas adjacent non-tumor tissues, precancerous lesions, and other non-malignant samples were excluded. Raw FASTQ files were uniformly processed using Cell Ranger (v8.0.1, 10x Genomics). Reads were aligned to the GRCh38 reference genome (refdata-gex-GRCh38-2020-A), followed by barcode processing and UMI quantification to generate gene-barcode matrices. The resulting matrices were analyzed using a unified downstream workflow in Scanpy (v1.10.3) for quality control, normalization, dimensionality reduction, batch correction, clustering, and visualization. Cells with fewer than 500 or more than 6,000 detected genes, or with more than 10% mitochondrial UMI content, were excluded. Doublets were removed using DoubletFinder (v2.0.2), and cells showing multi-lineage marker expression were further excluded. After normalization and Harmony-based batch integration, 197,812 high-quality cells were retained and clustered by uniform manifold approximation and projection (UMAP) into six major lineages: B/plasma, endothelial, epithelial, fibroblast, myeloid, and T/NK cells. Cell identities were annotated and validated using established canonical markers (30–33).

### Identification of T/NK, B and plasma cell subsets

Detailed subclustering was performed on T and B cell populations (34, 35). T/NK cells (n=75,218) were resolved into 13 transcriptionally distinct subsets: four CD4^+^ T cell states (naïve/TCM: IL7R^+^SELL^+^; Treg: FOXP3^+^; exhausted: TOX2^+^), seven CD8^+^ T cell states (naïve: THEMIS^+^; proliferating: MKI67^+^; tissue-resident memory: ZNF683^+^; effector: GZMK^+^; exhausted: PDCD1^+^; γδ T: TRDC^+^; MAIT: SLC4A10^+^), and two NK cell states (CD56^bright+^: TYROBP^+^; CD56^dim+^: FGFBP2^+^). B cells (n=37,450) were subdivided into nine subsets spanning naïve, memory, and germinal center states. Plasma cells (n=21,871) were partitioned into five subsets distinguished by immunoglobulin isotype and differentiation stage. Subset-defining marker expression was visualized using bubble plots and heatmaps.

### Pseudotime trajectory analysis

B cell differentiation trajectories were inferred using Slingshot and Monocle2. UMI counts were extracted from Seurat objects and imported into CellDataSet structures. Genes with mean expression > 0.1 were retained. DDRTree dimensionality reduction and orderCells trajectory ordering were subsequently applied.

### Spatial transcriptomic analysis

Spatial transcriptomic data from GSE251950 were analyzed in R using GSVA (version 2.6.2) and spacexr (version 1.5.0). TLS scores were calculated by ssGSEA using a 12-chemokine TLS signature, including *CCL2, CCL3, CCL4, CCL5, CCL8, CCL18, CCL19, CCL21, CXCL9, CXCL10, CXCL11*, and *CXCL13 (*36). ssGSEA was performed with a Gaussian kernel and default gene-set size parameters. Spatial spots with TLS score = 0 were defined as TLS-negative, whereas spots with TLS score > 0 were defined as TLS-positive. TLS-positive spots were further divided into TLS-low and TLS-high groups using the median TLS score as the cutoff. GSVA was then used to calculate B_C4_HSP70 and CD8T_C2_ZNF683 signature scores for each spatial spot. RCTD deconvolution was performed using spacexr with the integrated scRNA-seq atlas as the reference to infer B-cell enrichment across spatial spots.

### GSVA-based transcriptional signature scoring

Bulk RNA-seq data from TCGA-STAD and PRJEB25780 were transformed as log2(TPM + 1), converted from Ensembl IDs to gene symbols, and averaged for duplicated symbols. B_C4_HSP70 and CD8T_C2_ZNF683 gene sets were derived from positively enriched cluster markers identified by FindAllMarkers (**Supplementary Tables 4–6**). Sample-level signature scores were calculated using GSVA (v2.6.2; Gaussian kernel). Scores were used for survival and correlation analyses in TCGA-STAD and for group comparisons in PRJEB25780. These scores represent transcriptional signatures rather than direct cell-abundance estimates.

### Statistical analysis

All statistical analyses were performed using R software (version 4.4.2). Continuous variables were compared using Student’s t-test or the Wilcoxon rank-sum test, whereas categorical variables were analyzed using the chi-square test or Fisher’s exact test. Multi-group comparisons were performed using the Kruskal–Wallis test. Correlations were assessed using Spearman’s correlation analysis. Overall survival was evaluated using Kaplan–Meier (KM)analysis and compared by the log-rank test. For survival analyses, median split was used unless the variable showed a zero-inflated distribution with the median equal to zero. In such cases, patients were stratified by presence versus absence of the corresponding TLS feature (0 vs >0). Cox regression analyses were used to identify prognostic factors. Restricted cubic spline (RCS) analysis was applied to assess the relationship between TLS composition and mortality risk. Model performance was evaluated using the concordance index (C-index) and time-dependent receiver operating characteristic (ROC) analysis. Two-sided *P* values < 0.05 were considered statistically significant. For transcriptomic analyses involving multiple comparisons, *P* values were adjusted using the Benjamini–Hochberg method.

## Results

### Altered spatial distribution and decreased maturation of TLSs in *H. pylori*-positive GC

**Figure 1:**
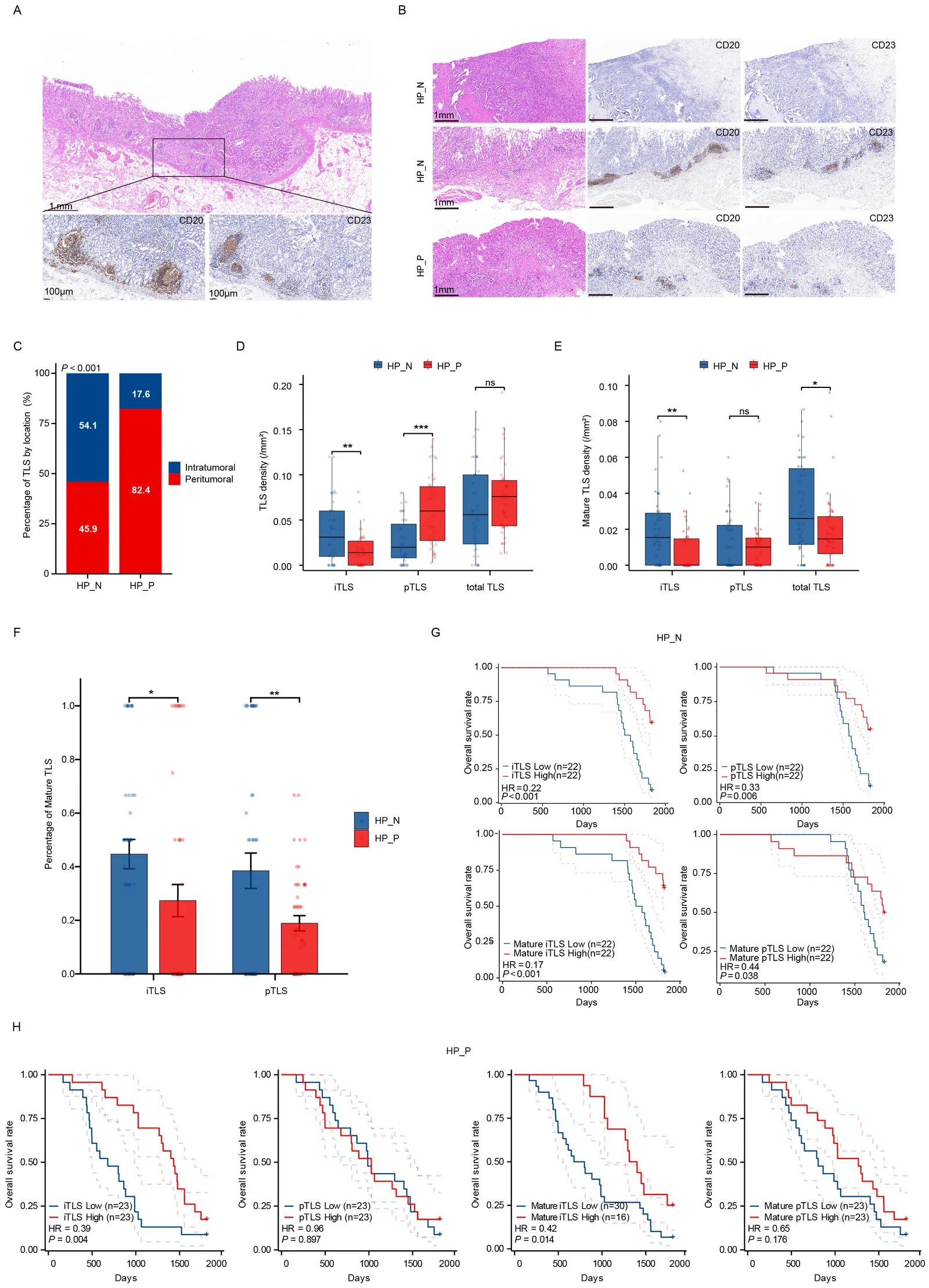
*H. pylori* status correlates with TLS distribution and maturation in GC. **(A)** Representative H&E staining and CD20/CD23 IHC staining of GC. Scale bars,1mm. Scale bars insets, 100μm. **(B)** Representative H&E staining and TLSs IHC staining of HP_P and HP_N GC. Scale bars, 1 mm. **(C)** Bar plot shows the proportional differences of TLS between intra-tumor and peri-tumor in the HP_N (n=44) and HP_P (n=46) groups. Chi-Square test. (**D–F**) Box plots show the iTLS, pTLS, and total TLS densities **(D)**; mature TLS densities **(E)**; and the proportions of mature iTLS and pTLS relative to total TLS between the HP_N and HP_P groups **(F)**. **(G, H)** KM analyses of overall survival according to iTLS density, pTLS density, mature iTLS density, and mature pTLS density in the HP_N **(G)** and HP_P **(H)** groups. For iTLS density, pTLS density, and mature pTLS density, patients were dichotomized at the group-specific median value. For mature iTLS density in the HP_P group, 30 of 46 patients had a value of zero; therefore, patients were categorized according to the presence (density > 0; n = 16) of mature iTLSs, rather than by a median split. Significant differences are determined using the Wilcoxon rank-sum test and log-rank test; ns: not significant, *p < 0.05, **p < 0.01, ***p < 0.001.

To define TLS spatial characteristics in relation to *H. pylori* status, we analyzed tumor tissues from 90 GC patients by H&E and IHC (**Supplementary Tables 1, 2**). TLSs (CD20^+^ B cell aggregates) were classified as immature lymphocyte aggregates or mature follicle-like structures based on CD23^+^ germinal center presence (12, 13) (**Figures 1A, B**). Chi-square analysis revealed significantly higher pTLS proportions in *H. pylori*-positive (HP_P) versus *H. pylori*-negative (HP_N) patients (**Figures 1B, C**), with density quantification confirming elevated pTLS in HP_P but elevated iTLS in HP_N patients (**Figure 1D**). HP_N patients also exhibited higher mature TLS densities intratumorally and overall (**Figure 1E**). Notably, the proportion of mature TLS (as a percentage of total TLS) was markedly reduced in both intratumoral and peritumoral regions of HP_P patients compared with HP_N patients (**Figure 1F**), suggesting an association between *H. pylori* status and impaired TLS maturation.

To further elucidate the prognostic significance of TLS spatial distribution and maturity in the context of *H. pylori* infection, KM analysis was performed and revealed opposing prognostic patterns. In HP_N patients, high TLS abundance (iTLS: HR = 0.22, *P* < 0.001; pTLS: HR = 0.33, *P* = 0.006) and high TLS maturity (iTLS: HR = 0.17, *P* < 0.001; pTLS: HR = 0.44, *P* = 0.038) were associated with favorable OS (**Figure 1G**). Conversely, in HP_P patients, iTLS (HR = 0.39, *P* = 0.004) retained favorable associations with OS, whereas pTLS (HR = 0.96, *P* = 0.897) abundance did not show prognostic benefit (**Figure 1H**). These findings indicate that the prognostic significance of TLS abundance and maturity in GC is highly dependent on *H. pylori* status, revealing distinct immune patterns between the two patient subgroups.

### ScRNA-seq landscape of *H. pylori*-stratified GC

**Figure 2:**
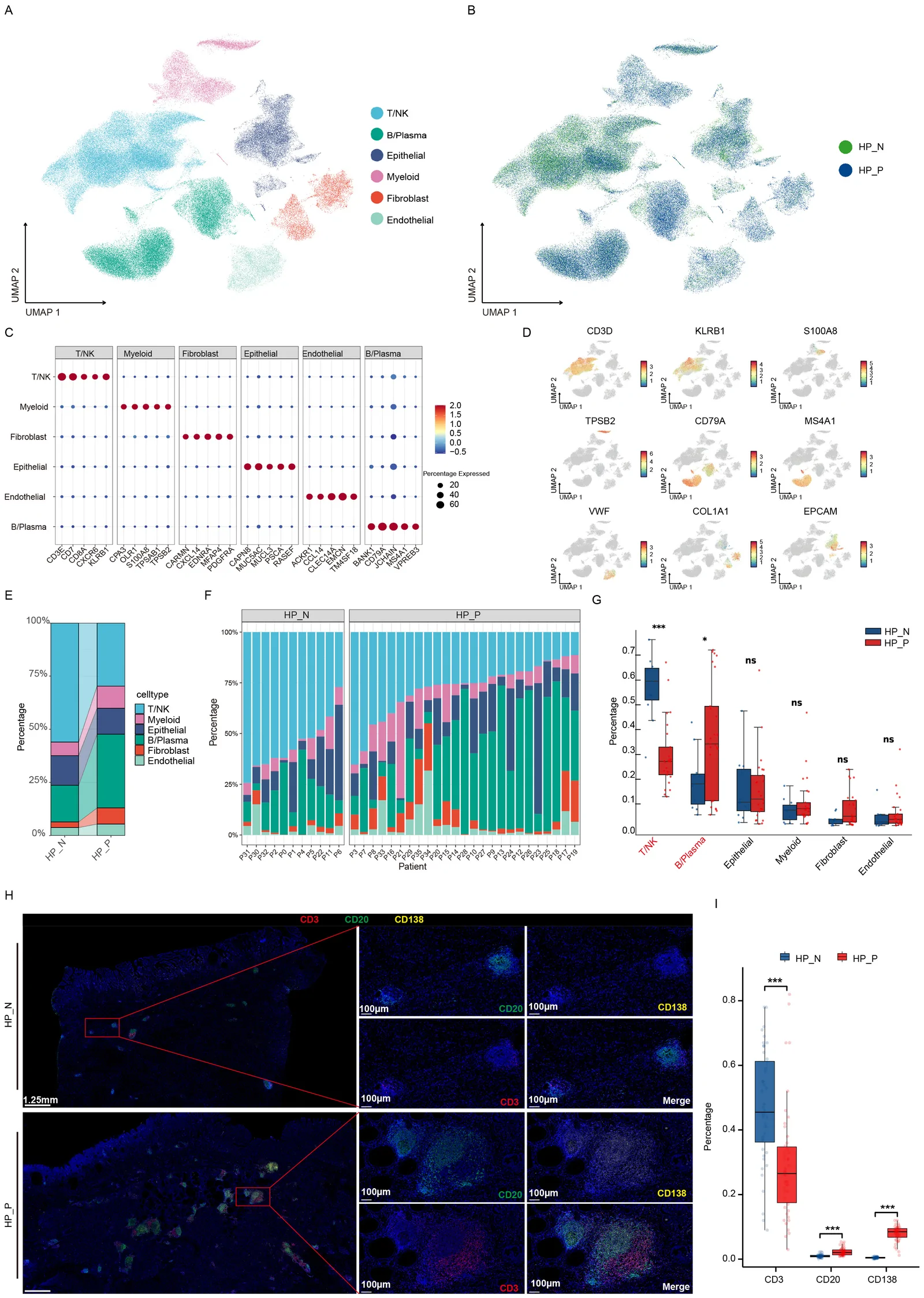
HP_P GC showed reduced T cells but elevated B/PCs. **(A, B)** UMAP plots showing the annotation and color codes by the cell clusters and group. **(C)** Dot plot of the expression level of top five marker genes for each cell cluster. **(D)** Feature plots showing the expression of canonical markers used to validate the major cell lineages: *CD3D* and *KLRB1* for T/NK cells, *S100A8* for myeloid cells, *TPSB2* for mast cells, *CD79A* and *MS4A1* for B/plasma cells, *VWF* for endothelial cells, *COL1A1* for fibroblasts, and *EPCAM* for epithelial cells. **(E–G)** Stacked bar charts and box plots comparing the proportions of major cell clusters between groups and patients. **(H)** Representative mIF images of CD20^+^ B cells (green), CD138^+^ plasma cells (yellow) and CD3^+^ T cells (red) of tumor tissues from HP_P and HP_N GC patients. Scale bars, 1.25 mm. Scale bars insets, 100μm. **(I)** Box plot revealing the proportions of CD3^+^, CD20^+^ and CD138^+^ cells between groups. Significant differences are determined using the Wilcoxon rank-sum test; ns: not significant, *p < 0.05, ***p < 0.001.

To dissect alterations in TIME in *H. pylori*-associated GC, we integrated public scRNA-seq data from HRA000704, GSE150290, and GSE249874 (**Supplementary Table 3**). The resulting atlas comprised 197,812 high-quality cells from 36 GC patients (25 HP_P, 11 HP_N) (**Supplementary Figures 1A, B**). UMAP clustering resolved six lineages, including B/plasma, endothelial, epithelial, fibroblast, myeloid, and T/NK cells (**Figure 2A**). These major lineages showed comparable distributions across groups, datasets, and patients, without apparent sample-specific clustering (**Figure 2B**; **Supplementary Figures 1C, D**). Dot plots highlighted the top five marker genes per lineage (**Figure 2C**), and canonical marker expression further validated the lineage identities (**Figure 2D**). Canonical markers included *CD3D* and *KLRB1* for T/NK cells, *S100A8* for myeloid cells, *TPSB2* for mast cells, *CD79A* and *MS4A1* for B/plasma cells, *VWF* for endothelial cells, *COL1A1* for fibroblasts, and *EPCAM* for epithelial cells. Furthermore, Silhouette analysis and repeated balanced subsampling further supported integration quality and reduced the likelihood that the major cell-compartment differences were solely driven by dataset origin or group-size imbalance (**Supplementary Figures 2A, B**). Notably, HP_P tumors displayed B/plasma cell expansion and T/NK cell reduction compared to HP_N tumors (**Figures 2E–G**), a pattern robust to inter-patient heterogeneity.

To validate these findings, we performed mIF on an independent GC cohort. HP_P specimens exhibited significantly elevated CD20^+^ B cell and CD138^+^ plasma cell proportions, with concomitant CD3^+^ T cell depletion (**Figures 2H, I**). These data highlight that *H. pylori*–associated GC exhibits a distinct immune landscape characterized by T-cell reduction and B/plasma cell expansion.

### HP_P GC exhibited significant depletion of ZNF683^+^CD8^+^ effector memory T cells

**Figure 3:**
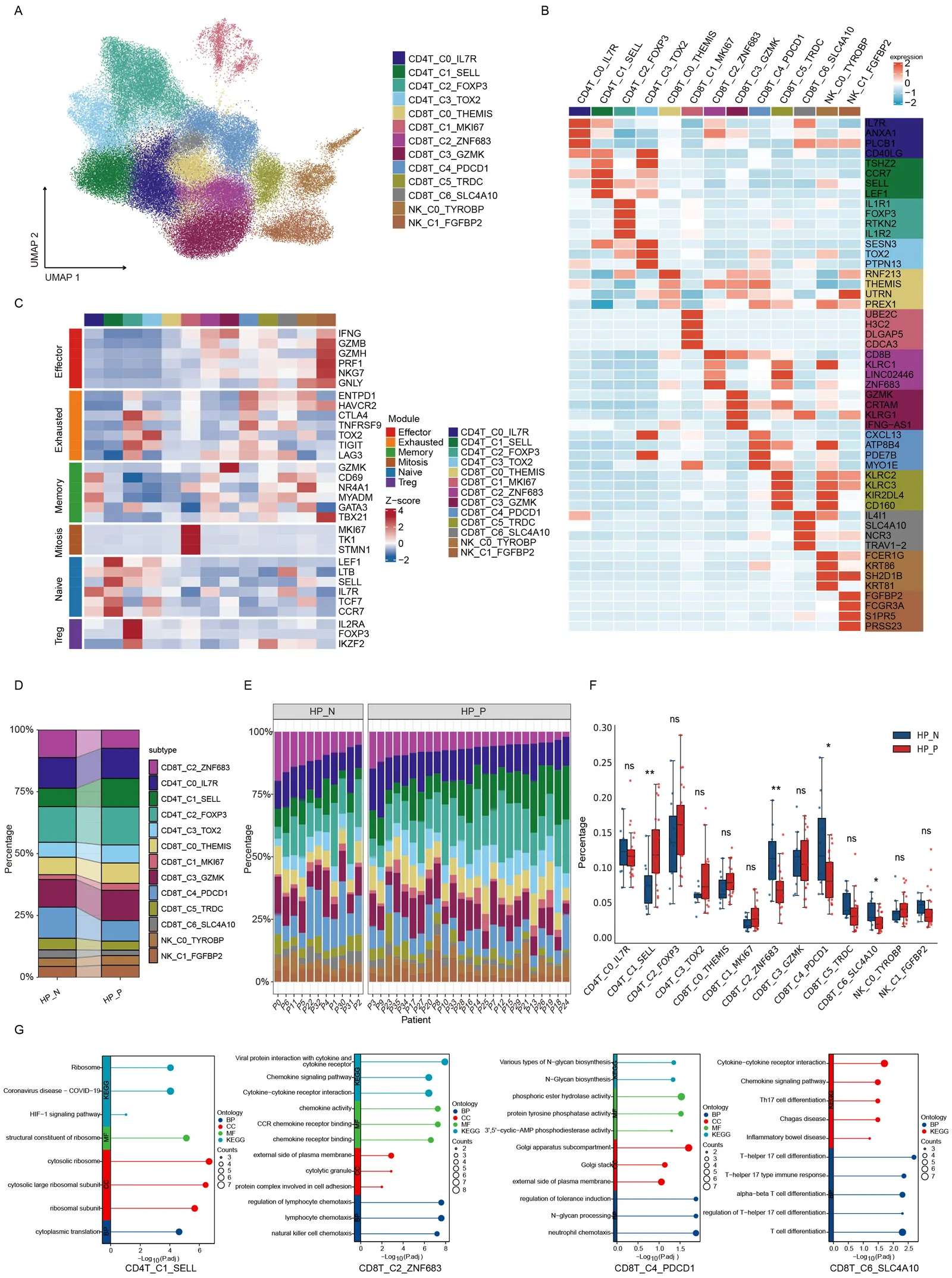
The number of CD8T_C2_ZNF683 cells is decreased in HP_P GC. **(A)** UMAP plot of T/NK cell clusters. **(B)** Heatmap showing the expression level of top four marker genes for each cell cluster. **(C)** Heatmap showing different functional module Z-score between T/NK cell clusters. **(D, E)** Proportional distribution of T/NK cell clusters across individual patients and groups. **(F)** Box plot showing the proportions of T/NK cell clusters in the HP_N and HP_P groups. **(G)** GO and KEGG enrichment of marker genes for CD4T_C1_SELL, CD8T_C2_ZNF683, CD8T_C4_PDCD1, and CD8T_C6_SLC4A10. Dot size indicates gene counts, and the x-axis shows −log_10_(adjusted *P* value). Significant differences are determined using the Wilcoxon rank-sum test; ns: not significant, *p < 0.05, **p < 0.01.

To systematically evaluate the effect of *H. pylori* infection on the infiltration and function of T cells in GC, unsupervised clustering identified 13 transcriptionally distinct subsets of 75,218 T/NK cells from GC specimens, including four CD4^+^ T cell states (CD4T_C0_IL7R, CD4T_C1_SELL, CD4T_C2_FOXP3, CD4T_C3_TOX2), seven CD8^+^ T cell states (CD8T_C0_THEMIS, CD8T_C1_MKI67, CD8T_C2_ZNF683, CD8T_C3_GZMK, CD8T_C4_PDCD1, CD8T_C5_TRDC, CD8T_C6_SLC4A10), and two NK cell states (NK_C0_TYROBP, NK_C1_FGFBP2) (**Figures 3A, B**; **Supplementary Figure 3A**). All clusters were consistently detected across groups and patients (**Supplementary Figures 3B, C**). Functional annotation revealed naïve (CD4T_C1_SELL), memory (CD4T_C0_IL7R), regulatory (CD4T_C2_FOXP3), and exhausted (CD4T_C3_TOX2) phenotypes in CD4^+^ compartments, naive (CD8T_C0_THEMIS), proliferating (CD8T_C1_MKI67), cytotoxic (CD8T_C2_ZNF683 and CD8T_C3_GZMK), and exhausted (CD8T_C4_PDCD1, CD8T_C5_TRDC and CD8T_C6_SLC4A10) phenotypes in CD8^+^ compartments and canonical cytotoxic NK cells (NK_C0_TYROBP and NK_C1_FGFBP2) (**Figure 3C**).

HP_P tumors exhibited selective expansion of naïve CD4^+^ T cells (CD4T_C1_SELL) and depletion of effector memory (CD8T_C2_ZNF683), exhausted (CD8T_C4_PDCD1), and MAIT-like (CD8T_C6_SLC4A10) CD8^+^ T cell subsets (**Figures 3D–F**; **Supplementary Figures 3D, E**). This pattern indicates broad disruption of CD8^+^ T cell differentiation and homeostasis, with pathway analysis confirming enrichment for ribosome and protein translation pathways in CD4T_C1_SELL, cytokine-cytokine receptor interaction, chemokine signaling and lymphocyte migration in CD8T_C2_ZNF683, N-glycosylation processing and immune regulation in CD8T_C4_PDCD1 and T cell differentiation and cytokine signaling pathways in CD8T_C6_SLC4A10 (**Figure 3G**). These findings demonstrate that T cell composition and functional status are systematically reshaped in HP_P GC, characterized by increased naive CD4^+^ T cells and decreased effector‑memory CD8^+^ T cell subsets.

### HP_P GC is characterized by increased HSP70^+^ B cells associated with poor prognosis

**Figure 4:**
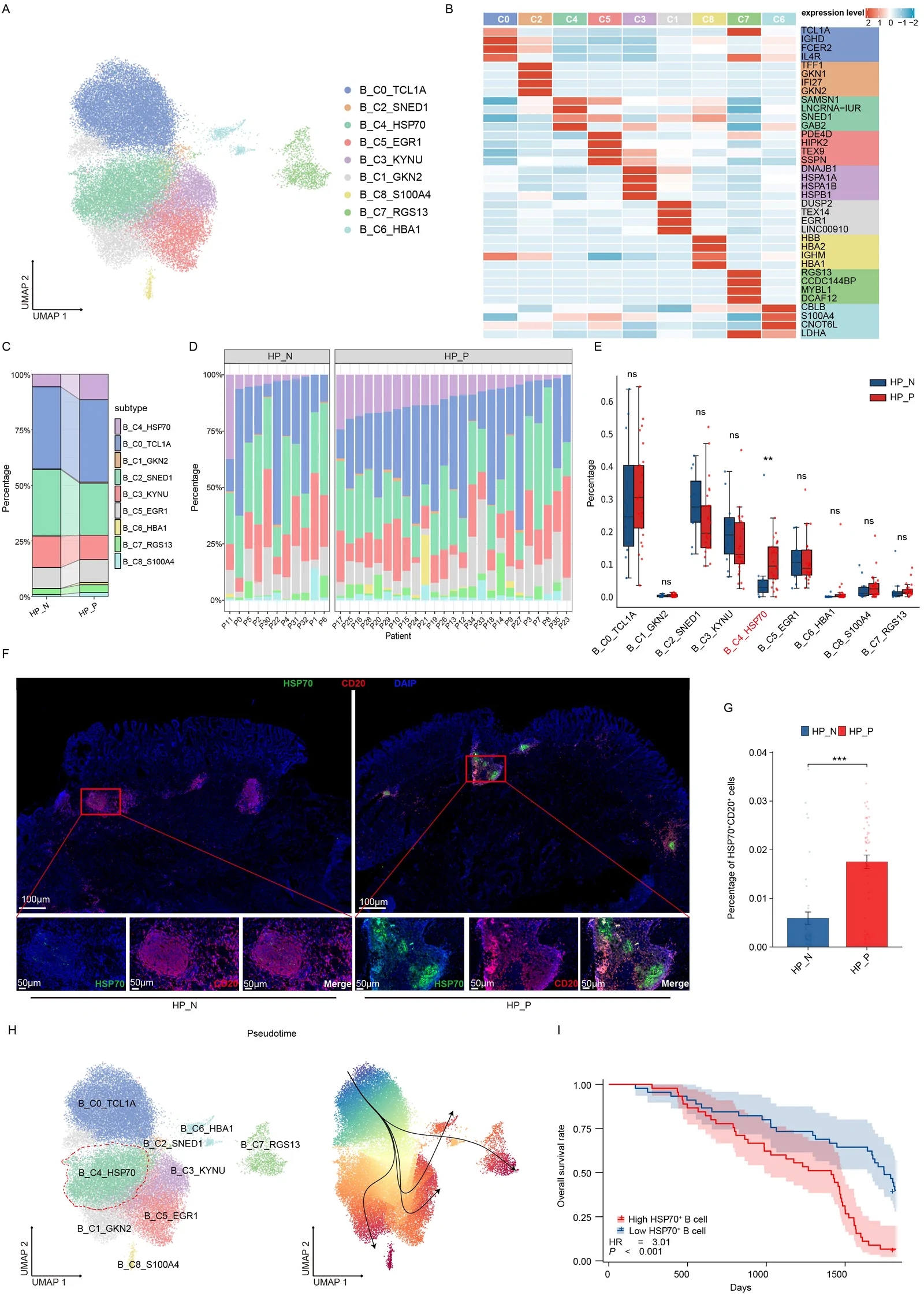
The differentiation landscape of B cells. **(A)** UMAP plot showing the subsets of B cells. **(B)** Heatmap of the Top4 marker genes for each B cell subcluster. **(C, D)** The distribution of B cell subclusters in all patients and between groups. **(E)** Box plot showing the relative proportions of B cell subgroups among groups. **(F)** Representative mIF images showing HSP70^+^ B cells in HP_N and HP_P GC tissues. HSP70 (green), CD20 (red). Scale bars, 100μm.Scale bars insets,50μm. **(G)** The density of HSP70^+^ B cells in HP_N and HP_P groups. **(H)** Pseudotime trajectory analysis of the B-cell lineage reveals four continuous differentiation. **(I)** KM analysis of OS stratified by HSP70^+^ B cells density. Significant differences are determined using the Wilcoxon rank-sum test and log-rank test; ns: not significant, **p < 0.01, ***p < 0.001.

A total of 37,450 B cells were resolved nine transcriptionally distinct subsets via unsupervised clustering, based on the expression profile of subset-specific marker genes (**Figures 4A, B**). All 9 subsets were stably detected across all patients, confirming the generalizability of this classification (**Supplementary Figures 4A, B**). Quantification of subset abundance showed that HSP70^+^ B cells were specifically expanded in the HP_P GC (**Figures 4C–E**; **Supplementary Figure 4C**). Notably, HSP70^+^ B cells represent a distinct cluster of stressed B cells typically generated under chronic environmental pressures (37), which aligns with the persistent inflammatory and oxidative stress characteristic of *H. pylori* infection. The mIF staining further confirmed significant enrichment of HSP70^+^ B cells in HP_P compared to HP_N GC patients (**Figures 4F, G**). GO functional annotation and KEGG pathway analysis confirmed the enrichment of protein folding and endoplasmic reticulum stress in HSP70^+^ B cells (**Supplementary Figure 4D**). Pseudotime analysis positioned HSP70^+^ B cells along a trajectory closer to GKN2^+^ B cells than to RGS13^+^ germinal-center-like B cells (**Figure 4H**; **Supplementary Figure 4E**). This pattern is consistent with previous evidence suggesting that HSP70^+^ B cells preferentially follow an extrafollicular rather than a germinal-center differentiation route (34).

Clinically, patients with high HSP70^+^ B-cell density had significantly shorter OS in the mIF cohort (HR = 3.01, *P* < 0.001; **Figure 4I**). Together, these findings link HSP70^+^ B-cell enrichment to an adverse prognostic phenotype in GC.

### Intra-TLS cellular composition is associated with predicted immunotherapy-related signatures

**Figure 5:**
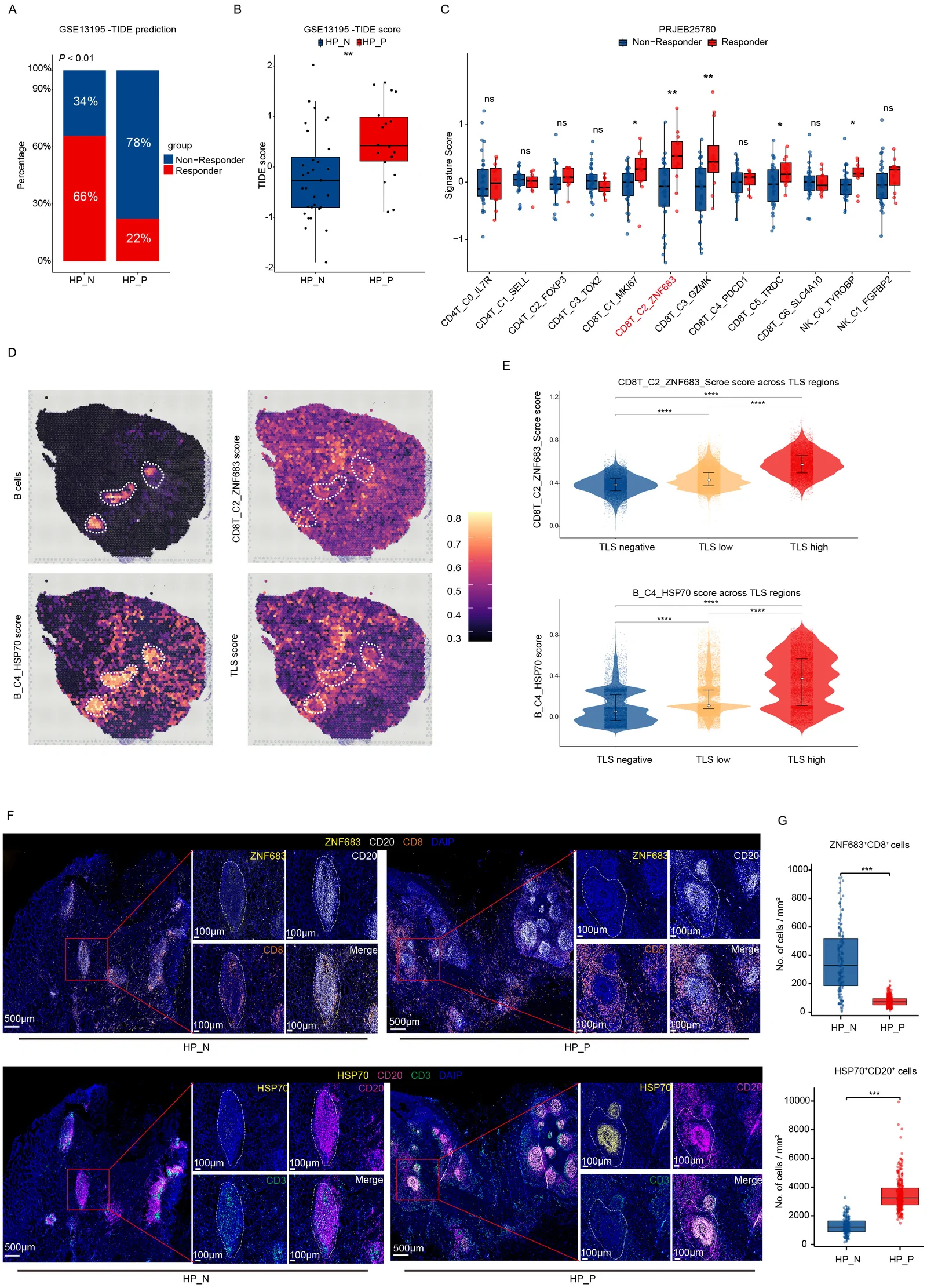
Spatial co-localization of ZNF683+CD8+ T cells and HSP70+ B cells within TLS. **(A)** TIDE-predicted response proportions in GSE13195 cohort. **(B, C)** Box plot of TIDE scores compared by HP_N and HP_P groups **(B)**; Box plot illustrating the scores of T/NK cell subclusters in responders and non-responders in the PRJEB25780 cohort **(C)**. **(D)** Representative ST section showing RCTD-inferred B-cell abundance and the spatial distribution of CD8T_C2_ZNF683, B_C4_HSP70, and TLS signature scores. **(E)** Violin plots showing CD8T_C2_ZNF683 and B_C4_HSP70 signature scores across TLS-negative, TLS-low, and TLS-high spatial spots. Each dot represents one spatial spot. **(F)** Representative mIF images showing ZNF683^+^CD8^+^ T cells and CD20^+^HSP70^+^ B cells in TLS regions of HP_N and HP_P GC tumor tissues. Scale bars, 500μm. Scale bars insets,100μm. **(G)** Box plots showing the densities of ZNF683^+^CD8^+^ T cells and HSP70^+^ B cells in HP_P and HP_N groups. Significant differences are determined using the Wilcoxon rank-sum test; ns: not significant, *p < 0.05, **p < 0.01, ***p < 0.001, ****p < 0.0001.

To evaluate the difference in potential immunotherapy response between HP_N and HP_P, TIDE algorithm analysis was performed (38). Of note, a superior projected immunotherapy response was found in HP_N compared to HP_P GC patients (66% vs. 22% response rates, *P* < 0.01; **Figure 5A**). Meanwhile, significantly elevated TIDE scores were found in the HP_P GC patients, suggesting a heightened potential for immune evasion (**Figure 5B**; **Supplementary Figure 5A**). Validation in PRJEB25780 dataset further confirmed that responders exhibited enriched cytotoxic signatures (ZNF683^+^, GZMK^+^, MKI67^+^, TRDC^+^ CD8^+^ T cells and TYROBP^+^ NK cells) (**Figure 5C**), but limited HSP70^+^ B cell signatures (**Supplementary Figure 5B**), contrasting with the HP_P immunosuppressive profile.

Subsequently, ST analysis of 9 GC tumor tissues showed that TLS-high regions spatially co-localized with RCTD-inferred B cell enrichment, ZNF683^+^CD8^+^ T-cell signatures, and HSP70^+^ B-cell signatures (**Figure 5D**; **Supplementary Figure 5C**). Quantitative comparison further showed that both ZNF683^+^CD8^+^ T-cell and HSP70^+^ B-cell signature scores were significantly higher in TLS-high regions than in TLS-low or TLS-negative regions (**Figure 5E**). These spot-level spatial findings were further supported by mIF, which confirmed the enrichment of HSP70^+^ B cells within TLS regions (**Supplementary Figure 5D**). Further quantification of these two cell populations within TLS regions showed that HSP70^+^ B cells were significantly enriched in TLSs from HP_P tumors, whereas ZNF683^+^CD8^+^ T cells were significantly increased in TLSs from HP_N tumors (**Figures 5F, G**). Together, these findings indicate that TLS regions contain both ZNF683^+^CD8^+^ T cell and HSP70^+^ B cell-associated signals, but the balance of these components differs by *H. pylori* status. Specifically, HP_P tumors show increased intra-TLS HSP70^+^ B-cell enrichment, whereas HP_N tumors show higher intra-TLS ZNF683^+^CD8^+^ T-cell representation. This compositional shift may contribute to the less immune-active, lower predicted immunotherapy-response profile observed in HP_P tumors.

### ZNF683^+^CD8^+^ T-cell reduction and HSP70^+^ B-cell expansion characterize an immature TLS compositional state in HP_P GC

**Figure 6:**
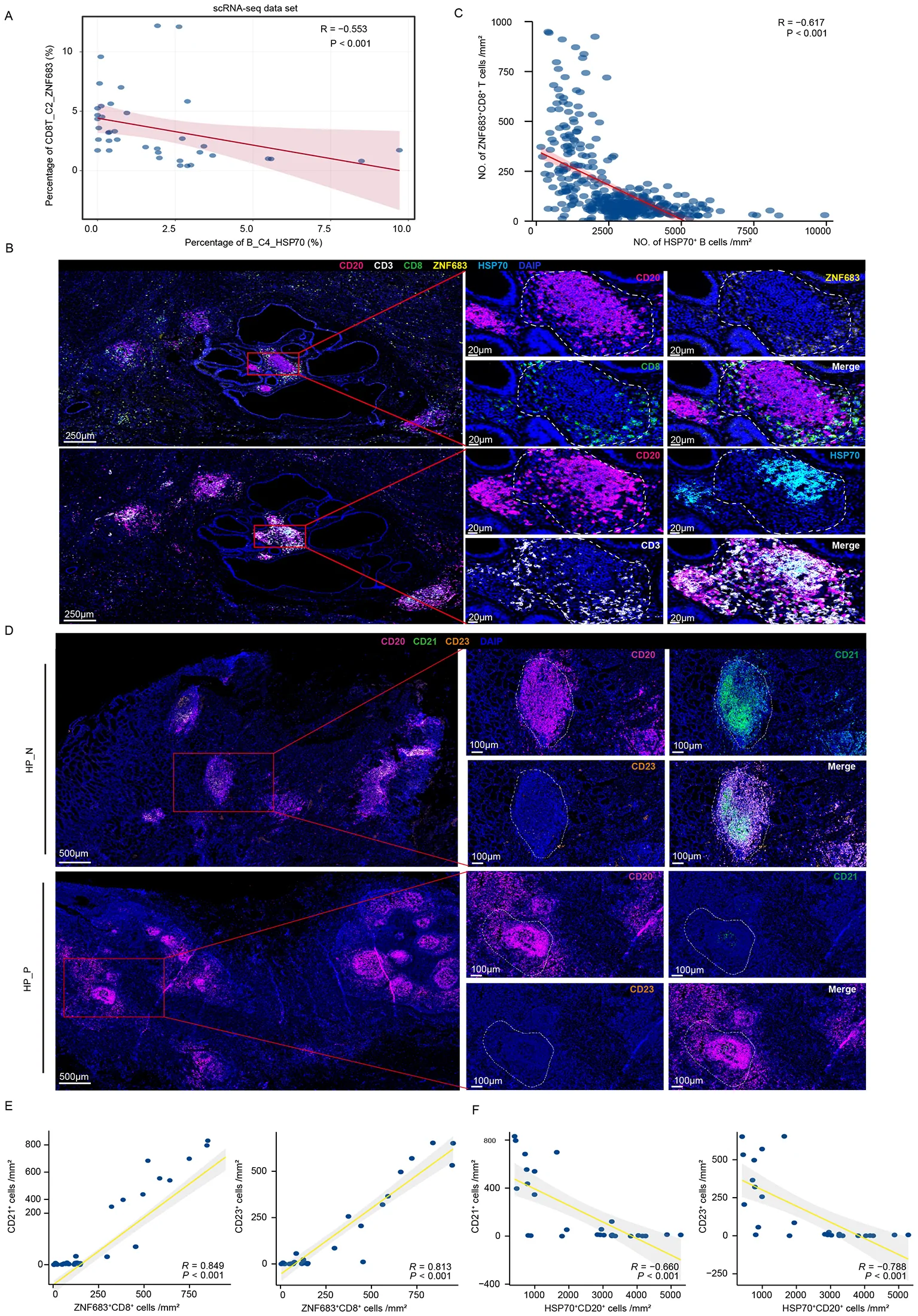
Germinal center and FDC network structures are compromised in HSP70+ B-cell-high TLSs of HP_P GC. **(A)** Scatterplot showing correlation between the proportions of HSP70^+^ B cell and ZNF683^+^CD8^+^ T cell in scRNA-seq data set. **(B)** Representative mIF images of CD20 (purple), CD3 (white), CD8 (green), HSP70 (cyan) and DAPI (blue) in HP_N and HP_P GC tumor tissues. Scale bars: 250μm and 20μm. **(C)** Scatterplot showing the correlation of HSP70^+^ B cell and ZNF683^+^CD8^+^ T cell densities in mIF cohort. **(D)** Representative mIF images of CD20 (purple), CD21(green), CD23 (orange) and DAPI (blue) in HP_N and HP_P GC tumor tissues. Scale bars, 500μm. Scale bars insets, 100μm. **(E, F)** Correlation analyses between the density of ZNF683^+^CD8^+^ T cells and CD21^+^/CD23^+^ cells, as well as between the density of HSP70^+^CD20^+^ B cells and CD21^+^/CD23^+^ cells. Spearman correlation test.

Correlation analysis of cell proportions based on scRNA-seq data further revealed a negative association between ZNF683^+^CD8^+^ T cells and HSP70^+^ B cells (R=-0.553, *P* < 0.001) (**Figure 6A**). In line with this observation, mIF analysis further showed a significant negative correlation between the intra-TLS densities of HSP70^+^ B cells and ZNF683^+^CD8^+^ T cells (R = -0.617, *P* < 0.001) (**Figures 6B, C**).

To assess TLS structural maturity, FDC networks and germinal centers were further evaluated. HP_N GC exhibited organized CD20^+^ B-cell follicles with continuous CD21^+^ FDC networks and CD23^+^ germinal center-like structures, whereas HP_P GC showed fragmented FDC networks and impaired germinal center-like organization (**Figure 6D**). Correlation analysis further supported these structural differences. ZNF683^+^ CD8^+^ T cell density was positively correlated with CD21^+^ and CD23^+^ signals, whereas HSP70^+^ B cell density showed inverse correlations with both markers (**Figures 6E, F**). Local *H. pylori* density was also positively correlated with intra-TLS HSP70^+^ B cell density (R = 0.7, *P* = 0.0073) (**Supplementary Figures 6A, B**). These findings suggest HP_P GC harbors a structurally aberrant TLS state characterized by HSP70^+^ B-cell accumulation, ZNF683^+^ CD8^+^ T-cell reduction, and disrupted FDC networks and germinal center-like structures.

### TLS composition refines prognostic stratification in HP_P GC

To further delineate the effect of *H. pylori* infection on TLS in GC, we classified TLS into two functional subtypes using K-means unsupervised clustering, based on the densities of HSP70^+^ B cells and ZNF683^+^CD8^+^ T cells within TLS (TLS ^type1^: HSP70^+^ B cell ^high^ ZNF683^+^CD8^+^T cell ^low^ subtype; TLS ^type2^: ZNF683^+^CD8^+^T cell ^high^ HSP70^+^ B cell ^low^ subtype).

**Figure 7:**
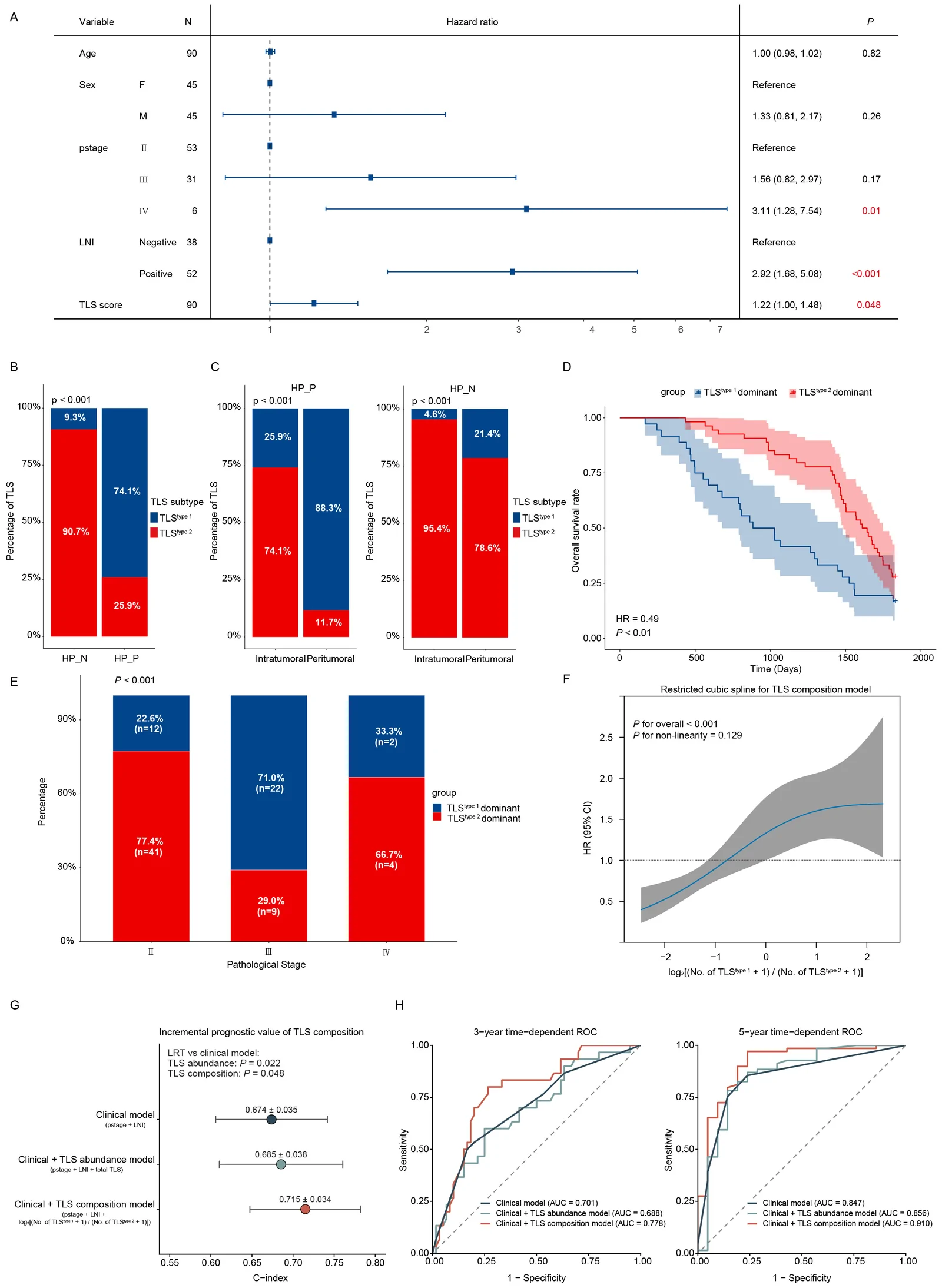
TLS subtype composition is associated with the clinical and prognostic features of gastric cancer. **(A)** Multivariable Cox regression analysis of OS incorporating clinicopathological variables and the TLS composition score, defined as log_2_[(TLStype^1^ + 1)/(TLStype^2^ + 1)]. **(B)** Proportions of TLS^type1^ and TLS^type2^ in HP_N and HP_P tumors. **(C)** Intratumoral and peritumoral distribution of TLS subtypes according to *H. pylori* status. **(D)** KM analysis stratified by dominant TLS subtypes; TLS^type1^ (n=52) vs. TLS^type2^ (n=38). **(E)** Distribution of TLS subtype dominance across pstages. **(F)** RCS showing the relationship between TLS composition and mortality risk. **(G)** C-index comparison showing the incremental prognostic value of TLS abundance and TLS composition beyond the clinical model. **(H)** Time-dependent ROC curves for 3-year and 5-year OS prediction. Significant differences are determined using the Wald test in the multivariable Cox model **(A)**, two-sided Fisher’s exact test **(B, C and E)**, log-rank test **(D)**, Wald tests for the RCS model **(F)**, and likelihood-ratio tests **(G)**.

Multivariable Cox regression identified pstage IV and positive LNI as adverse prognostic factors, whereas a higher TLS composition score, defined as log_2_[(TLS ^type1^ + 1)/(TLS ^type2^ + 1)], was independently associated with poorer OS, supporting a relative predominance of TLS ^type2^ over TLS^type1^ as a favorable prognostic feature (**Figure 7A**; **Supplementary Figures 7A, B**). Further analysis showed that *H. pylori* status was closely associated with TLS subtype composition. HP_N GC was predominantly characterized by TLS^type2^, whereas HP_P showed significant enrichment of TLS ^type1^ (**Figure 7B**). Spatial distribution analysis revealed that, in HP_P GC, TLS ^type1^ enrichment mainly occurred in the peritumoral region; by contrast, TLS ^type2^ predominated in both intratumoral and peritumoral regions in HP_N GC (**Figure 7C**). However, the distances from *H. pylori*-positive signals to TLS^type1^ and TLS^type2^ were not significantly different (**Supplementary Figure 7C**), suggesting that the association between *H. pylori* infection and TLS compositional remodeling may not be explained by direct local proximity alone.

We next evaluated the clinical relevance of TLS composition by stratifying patients according to TLS dominance (**Supplementary Figure 7D**). Patients with TLS ^type2^-dominant tumors had significantly better OS (**Figure 7D**), and the TLS ^type2^-dominant pattern was more frequently observed in stage II than in stage III disease (**Figure 7E**). RCS showed that the risk of death gradually increased with a higher TLS composition score, with a significant overall association (**Figure 7F**). In addition, after TLS composition was incorporated into a clinical model consisting of pstage and LNI, the model C-index further increased (**Figure 7G**) and the highest AUCs were achieved in the 3-year and 5-year time-dependent ROC analyses, suggesting that TLS composition provides stronger incremental prognostic value than TLS abundance alone (**Figure 7H**; **Supplementary Figure 7E**). These results highlight TLS composition as a clinically relevant immune structural biomarker that can further improve prognostic stratification beyond conventional clinicopathological factors and simple TLS quantity.

In conclusion, the immune cellular composition within TLSs is extensively remodeled in HP_P GC,and these alterations are closely associated with clinical outcomes in GC patients. Notably, the TLS composition represents a promising independent biomarker for prognostic and risk stratification in GC (**Graphical Abstract**).

## Discussion

In this study, we integrated scRNA-seq, ST, and mIF to characterize TLS remodeling in *H. pylori*-associated GC. *H. pylori*-positive tumors showed altered TLS localization, reduced TLS maturation, enrichment of HSP70^+^ B cells and reduced representation of ZNF683^+^CD8^+^ T cells within TLSs. These compositional changes were accompanied by disorganized FDC networks and germinal-center-like structures, as well as distinct clinical associations. Previous studies across multiple cancer types have shown that TLS abundance, spatial distribution, and maturation status are closely associated with patient prognosis (12–18). However, the extent to which the cellular composition of TLS modifies its prognostic relevance remains insufficiently explored, particularly in *H. pylori*-positive GC (39–41). Our findings suggest that the relative abundance of HSP70^+^ B cells and ZNF683^+^CD8^+^ T cells within TLSs is associated with TLS maturation, lymphoid architecture, and clinical outcome. Specifically, HSP70^+^ B-cell-enriched TLSs were associated with a less mature structural state, whereas TLSs with greater ZNF683^+^CD8^+^ T-cell representation were associated with more preserved FDC and germinal-center-like organization.

ZNF683, also known as Hobit, is a transcription factor associated with tissue-resident memory and cytotoxic T cell programs (42, 43). In our analysis, ZNF683^+^CD8^+^ T cells were reduced in *H. pylori*-positive tumors and were positively associated with mature TLS-related structures, suggesting that this subset may mark an immune-active TLS state.

HSP70 is a stress-responsive chaperone involved in protein folding and cellular adaptation to stress (44–47), and previous studies have reported HSP70-related B-cell states with immunomodulatory or extrafollicular features (37, 48). Using mIF, we confirmed the presence of a B-cell population with high HSP70 expression in GC tumor tissues. HSP70^+^ B cells were enriched in *H. pylori*-positive tumors and accumulated within TLSs displaying less organized FDC networks and germinal-center-like architecture. However, whether this B-cell state is directly induced by *H. pylori* infection remains to be determined. It also remains unclear whether HSP70^+^ B cells actively contribute to TLS immaturity or instead reflect the altered inflammatory context of *H. pylori*-positive tumors. Notably, *H. pylori*-positive signals were not significantly closer to TLS ^type1^ than to TLS ^type2^. Together with the predominant localization of *H. pylori* at the gastric mucosal surface (49, 50), this finding suggests that the observed TLS compositional remodeling may not be explained by direct bacterial proximity alone, but may instead reflect a broader *H. pylori*-associated inflammatory milieu.

Based on these two intra-TLS immune components, we defined two TLS compositional states. TLS ^type1^, characterized by enrichment of HSP70^+^ B cells and reduced representation of ZNF683^+^CD8^+^ T cells, was preferentially observed in *H. pylori*-positive tumors. TLS ^type2^, characterized by higher ZNF683^+^CD8^+^ T-cell representation and lower HSP70^+^ B-cell abundance, was more frequently observed in *H. pylori*-negative tumors and was associated with favorable OS. Incorporating TLS composition into the clinicopathological model further improved prognostic performance, suggesting that TLS compositional profiling, particularly the protective TLS ^type2^ feature, may provide additional prognostic information beyond conventional pathological factors and TLS abundance alone.

These findings may also be relevant to immunotherapy stratification. *H. pylori* infection has been linked to immune modulation and reduced benefit from ICI in GC (9–11). In our analysis, *H. pylori*-positive tumors showed reduced T/NK cell representation, enrichment of B/plasma cell compartments and higher inferred immune evasion scores. In external transcriptomic analyses, immune signatures corresponding to ZNF683^+^CD8^+^ T cells were enriched in responders, whereas HSP70^+^ B-cell signatures showed the opposite tendency. These results suggest that the TLS compositional state identified here may be linked to immunotherapy-related immune activity. However, this conclusion is based on computational prediction and external transcriptomic signatures rather than direct clinical response data from *H. pylori*-stratified ICI-treated patients. Therefore, the immunotherapy relevance of these TLS states should be considered hypothesis-generating.

This study has several limitations. First, the clinical cohort was retrospective and relatively limited in size, and the prognostic value of TLS composition requires validation in larger independent cohorts. Second, although Silhouette coefficient analysis and repeated balanced subsampling supported the robustness of the integrated single-cell results, the public scRNA-seq datasets differed in cohort composition and *H. pylori* distribution, which may have introduced dataset-specific confounding. Third, although our multi-omics and spatial analyses support an association between *H. pylori* status and TLS remodeling, they do not establish causality. Functional studies are needed to determine whether *H. pylori* directly promotes HSP70^+^ B-cell enrichment, reduces ZNF683^+^CD8^+^ T-cell representation, or impairs TLS maturation. Finally, the mechanisms through which *H. pylori* interacts with B- and T-cell compartments and influences TLS cellular composition, spatial organization, and functional maturation remain to be elucidated.

In summary, our study links *H. pylori*-positive gastric cancer to a distinct TLS compositional state characterized by HSP70^+^ B-cell enrichment, reduced ZNF683^+^CD8^+^ T-cell representation and less mature lymphoid organization. These findings highlight the importance of TLS quality and cellular composition in gastric cancer and suggest that TLS compositional profiling may refine prognostic stratification beyond TLS enumeration alone.

## Conclusion

This study characterizes immune alterations associated with *H. pylori* in GC, linked to altered T- and B-cell homeostasis and TLS remodeling. It also identifies a distinct TLS pattern that may serve as a candidate correlate for prognostic stratification and provide preliminary insights into immunotherapeutic strategies in *H. pylori*-positive GC.

## Data Availability

The original contributions presented in the study are included in the article/**Supplementary Material**. Further inquiries can be directed to the corresponding authors.
