# Supplementary material for: Single-cell and spatial landscape reveals impaired tertiary lymphoid structure maturation in Helicobacter pylori–associated gastric cancer
Source: Front Immunol. 2026 Aug 13;17:1908868. doi: 10.3389/fimmu.2026.1908868 (PMC13518559; doi:10.3389/fimmu.2026.1908868)
Supplement: Supplementary file 1 [file SupplementaryFile1.doc]

**Supplementary files**

**Single-cell and spatial landscape reveals impaired tertiary lymphoid structure maturation in *Helicobacter pylori*–associated gastric cancer**

Nenglin Zhang^1*^, Qiuyu Jiang^2*^, Qian Ma^1*^, Haisheng Huang^4^, Lijie Ma^3#^, Shuping Zhou^1#^

**Author affiliation:**

^1^Department of Gastroenterology, The First Affiliated Hospital of Anhui University of Science and Technology, Huainan, 232000, China;

^2^Department of Gastroenterology and Hepatology, Shanghai Institute of Liver Diseases, Zhongshan Hospital of Fudan University, Shanghai, 200032, China;

^3^Department of General Surgery, Zhongshan Hospital, Fudan University, Shanghai, 200032, China;

^4^Department of Dermatology, The Children’s Hospital of Fudan University, Shanghai, 201102, China;

* These authors contributed equally to this work.

^#^ Correspondence: 15821523323@163.com (L.M.), fy2019088@aust.edu.cn (S.Z.).

**Contents**

- **Supplementary results**
- **Supplementary materials and methods**
- **Supplementary figures**

**Fig. S1** Overview landscape of all cell populations in HP_P and HP_N GC.

**Fig. S2** Sensitivity analyses for integrated public single-cell datasets.

**Fig. S3** Analysis of T/NK cell subtypes in HP_P and HP_N samples.

**Fig. S4** Profiling and functional analysis of B cell subsets.

**Fig. S5** TIDE-related scores and B cell subcluster correlation analysis.

**Fig. S6** *H. pylori* burden is associated with HSP70^+^ B-cell enrichment.

**Fig. S7** TLS composition is associated with clinicopathological characteristics and prognostic model performance.

**Fig. S8** Analysis of Plasma cell subclusters.

- **Supplementary Tables**

**Supplementary Table 1** Clinical characteristics of GC cohort.

**Supplementary Table 2** Clinical characteristics stratified by *H. pylori* status (HP_N vs. HP_P) in GC patients.

**Supplementary Table 3** The clinical data of 36 GC patients in scRNA-seq cohort.

**Supplementary Table 4** Top10 marker genes of major cell clusters.

**Supplementary Table 5** Top10 marker genes of T/NK cell clusters.

**Supplementary Table 6** Top10 marker genes of B cell clusters.

**Supplementary results**

**Analysis of plasma cell subclusters.**

Five distinct plasma cell subsets (PC_C0, PC_C1, PC_C2, PC_C3, and PC_C4) were identified by UMAP clustering analysis (**Fig. S8A**). The Dotplot (**Fig. S8B**) shows the expression patterns of 15 marker genes across these five subsets. *IGKC* and *IGHG1/IGHG4* were broadly expressed across most subsets, suggesting shared plasma cell features. In contrast, each subset displayed specific marker genes. PC_C0 showed high expression of *IGLV6-57* and *IGKV4-1*. PC_C1 was characterized by high expression of *FYB1*, *CD96*, and *FYN*. PC_C2 highly expressed *IGLV2-11*, *IGLV2-14*, and *IGLV2-28*. PC_C3 was enriched for *MED13L*, *CPEB4*, and *TRIO*. PC_C4 showed high expression of *SNRPB2* and *IGHG1*. These differences indicate molecular heterogeneity among plasma cell subsets. We further evaluated the distribution of these subsets between the HP_N and HP_P groups using boxplots (**Fig. S8C**). No significant differences in cell proportions were observed for any plasma cell subset. This result suggests that plasma cell composition is largely similar between HP_N and HP_P patients.

**Supplementary materials and methods**

**Multiplex immunofluorescence (mIF) staining and analysis**

Formalin-fixed and paraffin-embedded (FFPE) GC tissue samples were obtained from First Affiliated Hospital of Anhui University of Science and Technology. Detailed clinical information of the enrolled patients is provided in **Table S1**. The following primary antibodies were used in this study: anti-CD3 (Abcam, ab135372, 1:500), anti-CD20 (Abcam, ab9475, 1:200), anti-CD138(Abcam, ab130405, 1:200), anti-CD8 (Abcam, ab237709, 1:2000), anti-CD21 (Abcam, ab75985, 1:8000), anti-CD23 (Abcam, ab92495, 1:200), anti-HSP70 (Cell Signaling Technology, 46477S, 1:200), H. pylori (Abcam, ab172611,1:13000) and anti-ZNF683 (Thermo Fisher, PA5-54902, 1:500). FFPE tissue sections (4μm) were used for histopathological evaluation of GC patients. For mIF analysis, tissue sections were first baked in an oven at 60°C for 60 min, followed by deparaffinization in xylene, and rehydration through a graded series of ethanol solutions (100%, 95%, 70%). Subsequently, antigen retrieval was performed by microwave heating of the sections in Citrate buffer for 10 min. After 60 min of primary antibody incubation, the sections were incubated with secondary antibody at room temperature for 10 min. After each staining cycle, heat-induced epitope retrieval was performed to strip all antibodies, including primary and secondary antibodies. Then mIF staining was conducted using the Opal Polaris 7 Color Automation Detection Kit (NEL871001KT, Akoya Biosciences). Nuclei were counterstained with DAPI for 5 min, and the sections were coverslipped. Multispectral images were scanned using a ZEISS AXIOSCAN 7 slide scanner. Quantitative and localization analysis of target cells and regions of interest was performed using QuPath (v.0.5.1). Cell segmentation and marker thresholding were performed using automated algorithms, with minor manual adjustments to optimize cell boundary definition and exclude background noise. Positive staining thresholds were determined based on isotype control staining and negative tissue regions, with consistent settings applied across all samples.

**Differential expressed genes (DEGs) analysis**

DEGs were identified using the FindAllMarkers function in Seurat (v5.1.0) under R (v4.4.2). Differential expression analysis was performed using the Wilcoxon rank-sum test with assay = "RNA", slot = "data", test.use = "wilcox", min.pct = 0.25, logfc.threshold = 0.25, and only.pos = FALSE. *P* values were adjusted using the Benjamini–Hochberg method. Genes with an adjusted *P* value < 0.05 and an absolute average log2 fold change (|avg_log2FC|) > 1 were considered differentially expressed (**Table S4-6**).

**Gene functional enrichment analysis**

Gene Ontology (GO) and Kyoto Encyclopedia of Genes and Genomes (KEGG) enrichment analyses were performed in R (v4.4.2) using clusterProfiler (v4.14.6) and org.Hs.eg.db (v3.20.0). Cluster-specific marker genes identified by FindAllMarkers with an adjusted P value < 0.05 and |avg_log2FC| > 1 were used as input. Gene symbols were converted to Entrez identifiers using the bitr function. All genes tested in the corresponding differential-expression analysis were used as the background gene set. GO enrichment was performed using enrichGO. KEGG pathway enrichment was performed using enrichKEGG with organism = "hsa". For both analyses, *P* values were adjusted using the Benjamini–Hochberg method, and terms with an adjusted *P* value < 0.05 were considered significantly enriched.

**Tumor Immune Dysfunction and Exclusion (TIDE) Analysis**

TIDE scoring was performed using the GC transcriptome data of GSE13195 via the Cancer Immunology Data Engine (CIDE, https://cide.ccr.cancer.gov), to predict and compare the clinical response potential to immune checkpoint inhibitor (ICI) immunotherapy between *H. pylori-*positive and *H. pylori*-negative GC patients.

**Cox regression analysis**

Univariable and multivariable Cox proportional hazards regression analyses were performed to identify independent prognostic factors. Hazard ratios (HRs) and 95% confidence intervals (CIs) were calculated for each variable.

**Time-dependent ROC analysis**

Time-dependent receiver operating characteristic (ROC) curves were generated using the timeROC package to evaluate the predictive performance of different prognostic models. The area under the curve (AUC) was calculated at the specified time points.

**Concordance index analysis**

Harrell’s concordance index (C-index) was calculated to assess the discriminative ability of the prognostic models. C-index values were calculated for the clinical model, clinical plus TLS abundance model, and clinical plus TLS composition model. Higher C-index values indicate better predictive performance.

**Supplementary figures**

**Fig. S1** **Overview landscape of all cell populations in HP_P and HP_N GC.** (**A-B**) Quality control metrics across samples. Distribution of detected genes per cell (**A**) and total UMI counts per cell (**B**) shown for each sample. (**C-D**) UMAP plots showing the annotation and colour codes by datasets and patients.

**Fig. S2 Sensitivity analyses for integrated public single-cell datasets.** (**A**) Silhouette coefficient analysis of the integrated embedding. Annotated cell states showed higher silhouette scores than dataset origin and *H. pylori* status, whereas dataset origin and *H. pylori* status showed scores close to zero, suggesting that the global embedding structure was mainly organized by cellular identity rather than dataset source or *H. pylori* status.

(**B**) Repeated balanced subsampling analysis of major cell-compartment proportions. Equal numbers of HP_N and HP_P samples were randomly selected across 1,000 iterations, and the median proportional difference between HP_P and HP_N was calculated for each iteration. Values greater than zero indicate higher proportions in HP_P, whereas values less than zero indicate higher proportions in HP_N. T/NK cells showed consistently negative differences, indicating reduced T/NK proportions in HP_P samples after controlling for group-size imbalance, while B/Plasma cells showed predominantly positive differences.

**Fig. S3 Analysis of T/NK cell subtypes in HP_P and HP_N samples. (A)** The expression of marker genes in different clusters. (**B-C**) UMAP plots showing the annotation and colour codes by patients and groups. **(D)** Bar plot showing the number of cells assigned to each T/NK subtype in the HP_N and HP_P groups. **(E)** Density maps of cells projected onto the UMAP for the HP_N and HP_P groups. Color intensity indicates local cell density.

**Fig. S4 Profiling and functional analysis of B cell subsets.** (**A-B**) UMAP plots showing the annotation and colour codes by patients and groups. (**C**) Bar plots showing the number of cells assigned to each B cell subtype in the HP_N and HP_P groups. (**D**) GO and KEGG enrichment of the B_C4_HSP70 signature genes. The x-axis shows enrichment significance as −log_10_(adjusted P value); dot size indicates gene counts, and colors denote ontology terms (BP, CC, MF) and KEGG pathways. (**E**) UMAP plots showing the annotation and colour codes by pseudotime and B cell subsets.

**Fig. S5 TIDE-related scores and B cell subclusters correlation analysis.** (**A**) Box plots showing the TIDE-related score by groups. (**B**) Box plots illustrating the proportions of B cell subclusters in responders and non-responders in the PRJEB25780 cohort. (**C**) Heatmaps illustrate spatial localization of B cell, TLS, CD8T_C2_ZNF683, and B_C4_HSP70 scores in GC tissues. Spatial transcriptomic scores are color-coded. (**D**) Bar graphs quantify the distinct densities of HSP70^+^ B cells within TLS areas versus out TLS tissue regions. Significant differences are determined using the Wilcoxon rank-sum test; ns: not significant, *p < 0.05, **p < 0.01, ***p < 0.001.

**Fig. S6 *H. pylori* burden is associated with HSP70^+^ B-cell enrichment.** (**A**) Representative mIF image showing the spatial distribution of *H. pylori*, CD20^+^, CD21^+^, CD23^+^, and HSP70^+^ B cells in GC tissue. Scale bars 500μm. Scale bars insets,100μm. (**B**) Correlation analysis between *H. pylori* density and TLS-associated cellular features. *H. pylori* density was positively correlated with HSP70^+^ B cell density, whereas the correlation with CD21^+^ and CD23^+^ B cell density was not statistically significant. Correlations were assessed using Spearman correlation analysis.

**Fig. S7 TLS composition is associated with clinicopathological characteristics and prognostic model performance.** (**A**) KM survival curves comparing OS between TLS ^type2^-high and TLS ^type2^-low patients. Statistical significance was determined using the log-rank test. (**B**) Pie charts showing the distribution of TLS composition across pathological stage. (**C**) Ridge plot illustrates the distances from *H. pylori* to TLS ^type1^ and TLS ^type2^. (**D**) Sankey diagram illustrating the relationships among TLS composition, *H. pylori* status, lymph node invasion (LNI), and pathological stage (pstage). (**E**) Three- and five-year calibration curves of the clinical model, clinical + TLS abundance model, and clinical + TLS composition model.

**Fig. S8 Analysis of Plasma cell subclusters.** (**A**) UMAP of subclusters of plasma cells. (**B**) Dot plot showing the top3 marker genes of subclusters. (**C**) Box plot showing the proportion of plasma cell subclusters. Unpaired Wilcoxon test.

**Supplementary Tables**

**Table S1 Clinical characteristics of GC cohort.**

| **Patient** | **Sex** | **Age** | **pstage** | **Tumor size (cm)** | **LNI** | ***H. pylori*** | **OS** | **OS.TIME**  **(Days)** |
| --- | --- | --- | --- | --- | --- | --- | --- | --- |
| P01 | F | 39 | Ⅲ | 7.5×7.5×2 | 1 | 1 | 1 | 168 |
| P02 | M | 50 | Ⅱ | 10×7.5×1 | 0 | 1 | 0 | 1825 |
| P03 | M | 65 | Ⅱ | 4.5×3×1 | 0 | 0 | 0 | 1825 |
| P04 | F | 55 | Ⅱ | 2.5×2.3×1.5 | 0 | 0 | 0 | 1825 |
| P05 | F | 72 | Ⅱ | 7×5.5×2 | 0 | 0 | 0 | 1825 |
| P06 | M | 62 | Ⅱ | 7×6×2 | 0 | 0 | 0 | 1825 |
| P07 | M | 58 | Ⅱ | 4×4×1.5 | 0 | 0 | 0 | 1825 |
| P08 | F | 84 | Ⅱ | 3×2.5×0.5 | 0 | 0 | 0 | 1825 |
| P09 | M | 65 | Ⅱ | 6.5×4.5×1 | 0 | 0 | 0 | 1825 |
| P10 | F | 86 | Ⅱ | 7×6×1.5 | 0 | 1 | 0 | 1825 |
| P11 | M | 39 | Ⅲ | 6×5.5×0.3 | 1 | 1 | 1 | 799 |
| P12 | F | 60 | Ⅲ | 5×4×1 | 0 | 1 | 1 | 1028 |
| P13 | F | 26 | Ⅲ | 4×3.5×1 | 1 | 1 | 1 | 589 |
| P14 | M | 62 | Ⅲ | 15×13×11 | 0 | 1 | 0 | 1825 |
| P15 | F | 44 | Ⅲ | 2×2×1.5 | 0 | 1 | 1 | 1295 |
| P16 | F | 49 | Ⅳ | 6.5×6×1 | 0 | 0 | 1 | 1803 |
| P17 | M | 76 | Ⅲ | 3×2.1×0.4 | 1 | 1 | 1 | 496 |
| P18 | M | 19 | Ⅱ | 7×4×1.5 | 0 | 0 | 0 | 1825 |
| P19 | F | 77 | Ⅱ | 7×5.5×1 | 0 | 0 | 0 | 1825 |
| P20 | M | 45 | Ⅲ | 6×2.5×1.5 | 1 | 1 | 1 | 394 |
| P21 | F | 56 | Ⅱ | 9×6.5×2 | 0 | 0 | 0 | 1825 |
| P22 | M | 33 | Ⅱ | 2×1.5×0.4 | 0 | 0 | 0 | 1825 |
| P23 | M | 69 | Ⅱ | 3×2×0.5 | 0 | 0 | 0 | 1825 |
| P24 | F | 86 | Ⅱ | 3×2.8×1 | 0 | 0 | 0 | 1825 |
| P25 | M | 46 | Ⅱ | 11×8×2 | 1 | 0 | 0 | 1825 |
| P26 | F | 50 | Ⅱ | 1.8×1.5×1.2 | 1 | 0 | 0 | 1825 |
| P27 | F | 59 | Ⅲ | 4×4×1 | 1 | 0 | 1 | 1815 |
| P28 | F | 53 | Ⅱ | 5.5×4.5×3.5 | 1 | 1 | 1 | 1676 |
| P29 | F | 55 | Ⅱ | 7.5×5×1 | 1 | 0 | 1 | 1809 |
| P30 | F | 80 | Ⅱ | 3×2×0.6 | 1 | 1 | 1 | 246 |
| P31 | F | 49 | Ⅱ | 6×5.5×1 | 0 | 0 | 1 | 1789 |
| P32 | F | 50 | Ⅱ | 5.5×5×1 | 1 | 0 | 1 | 1689 |
| P33 | F | 43 | Ⅱ | 12×9×1.5 | 1 | 0 | 1 | 1710 |
| P34 | M | 55 | Ⅲ | 5×5×1 | 1 | 1 | 1 | 1448 |
| P35 | M | 59 | Ⅱ | 6×5×1.3 | 1 | 1 | 1 | 1746 |
| P36 | F | 63 | Ⅱ | 4.3×2.5×0.6 | 1 | 1 | 1 | 1027 |
| P37 | M | 61 | Ⅲ | 3×3×1 | 1 | 1 | 1 | 1064 |
| P38 | F | 60 | Ⅱ | 2.5×2.5×1 | 1 | 0 | 1 | 1748 |
| P39 | M | 44 | Ⅱ | 2.0×1.5×0.4 | 0 | 0 | 1 | 1642 |
| P40 | F | 29 | Ⅲ | 4×3×3 | 1 | 0 | 1 | 825 |
| P41 | M | 62 | Ⅲ | 10×8×2 | 1 | 1 | 1 | 973 |
| P42 | M | 75 | Ⅲ | 2.5×2×1 | 1 | 1 | 1 | 1401 |
| P43 | F | 51 | Ⅱ | 3.5×3×0.4 | 1 | 1 | 1 | 553 |
| P44 | F | 68 | Ⅱ | 8×7×1.5 | 0 | 0 | 1 | 1671 |
| P45 | M | 49 | Ⅲ | 5×4×1 | 1 | 1 | 1 | 1312 |
| P46 | F | 77 | Ⅲ | 15×10×10 | 1 | 0 | 1 | 654 |
| P47 | M | 62 | Ⅳ | 10×10×8.2 | 0 | 0 | 1 | 1456 |
| P48 | F | 81 | Ⅱ | 3×2×1 | 1 | 0 | 1 | 1650 |
| P49 | M | 56 | Ⅱ | 5×4.8×0.2 | 1 | 0 | 1 | 1610 |
| P50 | M | 50 | Ⅱ | 1.2×1.5×1.5 | 1 | 1 | 1 | 1478 |
| P51 | M | 48 | Ⅱ | 4.5×5×1.5 | 1 | 0 | 1 | 1600 |
| P52 | F | 72 | Ⅱ | 15×8×1.5 | 1 | 0 | 1 | 1724 |
| P53 | F | 58 | Ⅲ | 8×7×6 | 0 | 1 | 1 | 467 |
| P54 | M | 81 | Ⅱ | 6×6×5 | 1 | 0 | 1 | 1500 |
| P55 | F | 58 | Ⅱ | 4.5×3.5×1.2 | 1 | 1 | 1 | 884 |
| P56 | M | 50 | Ⅱ | 4×2×1 | 0 | 1 | 1 | 274 |
| P57 | F | 57 | Ⅲ | 5.5×3.5×1.5 | 1 | 1 | 0 | 1825 |
| P58 | M | 60 | Ⅱ | 3×2.5×1.5 | 0 | 0 | 1 | 1570 |
| P59 | M | 61 | Ⅱ | 8.5×7×5 | 1 | 0 | 1 | 1567 |
| P60 | F | 68 | Ⅲ | 1.8×1.7×0.2 | 0 | 1 | 1 | 1558 |
| P61 | F | 60 | Ⅲ | 7×4×1 | 1 | 1 | 1 | 1556 |
| P62 | M | 69 | Ⅲ | 3.5×2.2×0.6 | 0 | 1 | 0 | 1825 |
| P63 | M | 76 | Ⅲ | 8×7×6 | 0 | 0 | 1 | 1467 |
| P64 | M | 44 | Ⅲ | 7×5×4 | 1 | 1 | 1 | 863 |
| P65 | F | 33 | Ⅲ | 2.0×7×1.5 | 1 | 1 | 1 | 1523 |
| P66 | M | 74 | Ⅱ | 3.5×3×2 | 1 | 1 | 1 | 435 |
| P67 | F | 52 | Ⅱ | 3.5×2.5×0.5 | 1 | 1 | 1 | 807 |
| P68 | M | 57 | Ⅲ | 2.8×2.5×1 | 0 | 1 | 1 | 471 |
| P69 | M | 59 | Ⅱ | 3.5×3×1 | 0 | 1 | 1 | 679 |
| P70 | M | 66 | Ⅳ | 3.2×1.2×0.9 | 1 | 0 | 1 | 1484 |
| P71 | F | 67 | Ⅲ | 3×2×1.5 | 0 | 1 | 1 | 1472 |
| P72 | F | 66 | Ⅱ | 6×5.5×1.5 | 1 | 1 | 1 | 1267 |
| P73 | M | 74 | Ⅳ | 3.5×3×1 | 0 | 1 | 1 | 789 |
| P74 | M | 37 | Ⅲ | 5×4×1 | 1 | 1 | 1 | 442 |
| P75 | M | 71 | Ⅱ | 3.5×3×2 | 0 | 0 | 1 | 1465 |
| P76 | M | 76 | Ⅱ | 4×3×1.5 | 1 | 1 | 1 | 1028 |
| P77 | F | 82 | Ⅲ | 5×3×1.2 | 1 | 1 | 1 | 498 |
| P78 | F | 65 | Ⅱ | 4.5×2.5×0.3 | 0 | 0 | 1 | 1231 |
| P79 | M | 63 | Ⅳ | 20×13×1 | 1 | 1 | 1 | 649 |
| P80 | F | 27 | Ⅲ | 3.5×2×2 | 1 | 1 | 1 | 1433 |
| P81 | M | 84 | Ⅱ | 7×7×4 | 1 | 0 | 1 | 1502 |
| P82 | M | 61 | Ⅱ | 6×5×1.5 | 1 | 0 | 1 | 1427 |
| P83 | F | 59 | Ⅱ | 3.5×3.5×1.5 | 0 | 1 | 0 | 1825 |
| P84 | M | 68 | Ⅱ | 4×2×1 | 1 | 0 | 1 | 1174 |
| P85 | F | 71 | Ⅳ | 6×6×5 | 1 | 0 | 1 | 567 |
| P86 | F | 45 | Ⅲ | 3×2.5×1.7 | 1 | 0 | 1 | 1412 |
| P87 | F | 56 | Ⅱ | 4×3×2 | 1 | 0 | 1 | 1125 |
| P88 | M | 58 | Ⅲ | 5×3×1 | 1 | 1 | 1 | 986 |
| P89 | M | 51 | Ⅱ | 3×2×1.5 | 0 | 1 | 1 | 614 |
| P90 | F | 60 | Ⅱ | 2×2×0.8 | 0 | 1 | 1 | 987 |

pstage, Pathological stage; F, female; M, male; LNI, Lymph node invasion, 0: negative, 1: positive; *H. pylori*, 0: negative, 1: positive

**Table S2 Clinical characteristics stratified by *H. pylori* status (HP_N vs. HP_P) in GC patients.**

| **Variable** | **Level** | **Overall (n=90)** | **HP_N (n=44)** | **HP_P (n=46)** | **P_value** |
| --- | --- | --- | --- | --- | --- |
| Sex | F | 45 (50%) | 23 (52.3%) | 22 (47.8%) | 0.833* |
| Sex | M | 45 (50%) | 21 (47.7%) | 24 (52.2%) |  |
| Age |  | 59.5 (50-68) | 61 (50-71) | 58.5 (50-65.25) | 0.276^#^ |
| pstage | Ⅱ | 53 (58.9%) | 35 (79.5%) | 18 (39.1%) | <0.001* |
|  | Ⅲ | 31 (34.4%) | 5 (11.4%) | 26 (56.5%) |  |
|  | Ⅳ | 6 (6.7%) | 4 (9.1%) | 2 (4.3%) |  |
| Tumor size,  max diameter (cm) |  | 5 (3.28, 7) | 5.25 (3.15, 7) | 5.25 (3.15, 7) | 0.254^#^ |
| Lymph node invasion | Negative | 38 (42.2%) | 22 (50%) | 16 (34.8%) | 0.2* |
|  | Positive | 52 (57.8%) | 22 (50%) | 30 (65.2%) |  |

pstage, Pathological stage; HP_P, *H. pylori*-positive; HP_N, *H. pylori*-negative; Data are presented as median (Q1, Q3) for continuous variables and n (%) for categorical variables. *Tested by Fisher’s exact test; ^#^Tested by Wilcoxon rank-sum test.

**Table S3** The clinical data of 36 GC patients in scRNA-seq cohort.

| **Patient** | **Dataset** | **Sample** | **Cell counts** | **Group** |
| --- | --- | --- | --- | --- |
| P0 | HRA000704 | HRR207228 | 3888 | HP_N |
| P0 | HRA000704 | HRR207229 | 3319 | HP_N |
| P1 | HRA000704 | HRR207242 | 6240 | HP_N |
| P1 | HRA000704 | HRR207243 | 1958 | HP_N |
| P10 | GSE150290 | SRR11770302 | 5204 | HP_P |
| P11 | GSE150290 | SRR11770304 | 4008 | HP_N |
| P12 | GSE150290 | SRR11770306 | 5078 | HP_P |
| P13 | GSE150290 | SRR11770308 | 9995 | HP_P |
| P14 | GSE150290 | SRR11770312 | 4149 | HP_P |
| P15 | GSE150290 | SRR11770314 | 5831 | HP_P |
| P16 | GSE150290 | SRR11770316 | 5831 | HP_P |
| P17 | GSE150290 | SRR11770318 | 2400 | HP_P |
| P18 | GSE150290 | SRR11770320 | 5414 | HP_P |
| P19 | GSE150290 | SRR11770322 | 4596 | HP_P |
| P2 | HRA000704 | HRR207225 | 3112 | HP_N |
| P20 | GSE150290 | SRR11770324 | 3681 | HP_P |
| P21 | GSE150290 | SRR11770326 | 4558 | HP_P |
| P22 | GSE150290 | SRR11770328 | 4025 | HP_N |
| P23 | GSE150290 | SRR11770332 | 2848 | HP_P |
| P24 | GSE150290 | SRR11770334 | 1902 | HP_P |
| P25 | GSE150290 | SRR11770336 | 4967 | HP_P |
| P26 | GSE150290 | SRR11770338 | 3431 | HP_P |
| P27 | GSE150290 | SRR11770341 | 5112 | HP_P |
| P28 | GSE150290 | SRR11770343 | 6453 | HP_P |
| P29 | GSE150290 | SRR11770345 | 4616 | HP_P |
| P3 | HRA000704 | HRR207231 | 4902 | HP_P |
| P30 | GSE249874 | GSM7966226 | 7729 | HP_N |
| P31 | GSE249874 | GSM7966227 | 5622 | HP_N |
| P32 | GSE249874 | GSM7966228 | 3172 | HP_N |
| P33 | GSE249874 | GSM7966229 | 5800 | HP_P |
| P34 | GSE249874 | GSM7966230 | 6237 | HP_P |
| P35 | GSE249874 | GSM7966231 | 11416 | HP_P |
| P4 | HRA000704 | HRR207233 | 3926 | HP_N |
| P5 | HRA000704 | HRR207236 | 13902 | HP_N |
| P6 | HRA000704 | HRR207239 | 5546 | HP_N |
| P7 | HRA000704 | HRR207245 | 5837 | HP_P |
| P8 | HRA000704 | HRR207249 | 7943 | HP_P |
| P9 | GSE150290 | SRR11770300 | 3164 | HP_P |

HP_P, *H. pylori*-positive; HP_N, *H. pylori*-negative

**Table S4** Top10 marker genes of major cell clusters.

| avg_log2FC | pct.1 | pct.2 | p_val_adj | cluster | gene |
| --- | --- | --- | --- | --- | --- |
| 7.434788 | 0.26 | 0.003 | 0 | T/NK | CXCR6 |
| 7.368177 | 0.699 | 0.012 | 0 | T/NK | CD3E |
| 7.308877 | 0.311 | 0.006 | 0 | T/NK | CD8A |
| 7.109773 | 0.456 | 0.013 | 0 | T/NK | KLRB1 |
| 6.675364 | 0.622 | 0.019 | 0 | T/NK | CD7 |
| 6.570119 | 0.687 | 0.015 | 0 | T/NK | CD2 |
| 6.537193 | 0.498 | 0.011 | 0 | T/NK | ICOS |
| 6.448126 | 0.445 | 0.013 | 0 | T/NK | GZMA |
| 6.435293 | 0.353 | 0.009 | 0 | T/NK | LINC01871 |
| 6.419262 | 0.755 | 0.021 | 0 | T/NK | CD3D |
| 6.417768 | 0.282 | 0.006 | 0 | T/NK | PRF1 |
| 6.402566 | 0.262 | 0.005 | 0 | T/NK | CTLA4 |
| 6.38016 | 0.423 | 0.009 | 0 | T/NK | THEMIS |
| 6.342589 | 0.518 | 0.008 | 0 | T/NK | CD3G |
| 6.282228 | 0.287 | 0.005 | 0 | T/NK | ZAP70 |
| 9.631238 | 0.282 | 0.008 | 0 | Myeloid | TPSAB1 |
| 8.95939 | 0.293 | 0.02 | 0 | Myeloid | TPSB2 |
| 8.686012 | 0.27 | 0.005 | 0 | Myeloid | CPA3 |
| 8.494633 | 0.259 | 0.003 | 0 | Myeloid | HPGDS |
| 7.407316 | 0.335 | 0.008 | 0 | Myeloid | S100A8 |
| 7.194972 | 0.303 | 0.004 | 0 | Myeloid | OLR1 |
| 7.166577 | 0.306 | 0.002 | 0 | Myeloid | FPR1 |
| 6.981546 | 0.36 | 0.004 | 0 | Myeloid | CSF2RA |
| 6.922421 | 0.273 | 0.002 | 0 | Myeloid | LILRB2 |
| 6.891977 | 0.263 | 0.002 | 0 | Myeloid | FPR3 |
| 6.767848 | 0.272 | 0.003 | 0 | Myeloid | TREM1 |
| 6.736104 | 0.272 | 0.004 | 0 | Myeloid | AQP9 |
| 6.698501 | 0.262 | 0.003 | 0 | Myeloid | LILRB4 |
| 6.642987 | 0.451 | 0.019 | 0 | Myeloid | S100A9 |
| 6.591571 | 0.406 | 0.012 | 0 | Myeloid | CD14 |
| 7.6837 | 0.525 | 0.042 | 0 | Epithelial | MUC5AC |
| 7.622367 | 0.296 | 0.008 | 0 | Epithelial | MUCL3 |
| 7.445142 | 0.427 | 0.005 | 0 | Epithelial | RASEF |
| 7.303175 | 0.466 | 0.007 | 0 | Epithelial | CAPN8 |
| 7.297879 | 0.38 | 0.017 | 0 | Epithelial | PSCA |
| 7.153307 | 0.471 | 0.005 | 0 | Epithelial | LIPH |
| 7.134492 | 0.299 | 0.011 | 0 | Epithelial | MSMB |
| 7.059291 | 0.325 | 0.007 | 0 | Epithelial | CLDN3 |
| 7.033917 | 0.505 | 0.044 | 0 | Epithelial | TFF2 |
| 6.992136 | 0.497 | 0.143 | 0 | Epithelial | PGC |
| 6.985781 | 0.42 | 0.035 | 0 | Epithelial | REG1A |
| 6.938768 | 0.443 | 0.029 | 0 | Epithelial | PHGR1 |
| 6.926807 | 0.605 | 0.09 | 0 | Epithelial | TFF1 |
| 6.832753 | 0.794 | 0.024 | 0 | Epithelial | ELF3 |
| 6.82904 | 0.28 | 0.002 | 0 | Epithelial | EPS8L3 |
| 7.056324 | 0.387 | 0.005 | 0 | B/Plasma | VPREB3 |
| 6.482596 | 0.763 | 0.019 | 0 | B/Plasma | CD79A |
| 6.19816 | 0.503 | 0.027 | 0 | B/Plasma | BANK1 |
| 6.060007 | 0.456 | 0.014 | 0 | B/Plasma | MS4A1 |
| 5.936907 | 0.751 | 0.264 | 0 | B/Plasma | JCHAIN |
| 5.73182 | 0.613 | 0.232 | 0 | B/Plasma | IGLC2 |
| 5.727555 | 0.309 | 0.102 | 0 | B/Plasma | IGHG3 |
| 5.688632 | 0.402 | 0.108 | 0 | B/Plasma | IGLC3 |
| 5.678772 | 0.28 | 0.005 | 0 | B/Plasma | TNFRSF17 |
| 5.583036 | 0.472 | 0.129 | 0 | B/Plasma | IGHA2 |
| 5.509005 | 0.825 | 0.359 | 0 | B/Plasma | IGHA1 |
| 5.491207 | 0.617 | 0.116 | 0 | B/Plasma | IGHM |
| 5.320861 | 0.37 | 0.024 | 0 | B/Plasma | DERL3 |
| 5.304026 | 0.852 | 0.463 | 0 | B/Plasma | IGKC |
| 5.217883 | 0.41 | 0.029 | 0 | B/Plasma | ANGPTL1 |
| 8.914486 | 0.26 | 0.001 | 0 | Fibroblast | CARMN |
| 8.850397 | 0.368 | 0.002 | 0 | Fibroblast | MFAP4 |
| 8.576377 | 0.306 | 0.008 | 0 | Fibroblast | CXCL14 |
| 8.451808 | 0.314 | 0.003 | 0 | Fibroblast | PDGFRA |
| 8.357905 | 0.516 | 0.013 | 0 | Fibroblast | LUM |
| 8.337432 | 0.383 | 0.002 | 0 | Fibroblast | EDNRA |
| 8.326455 | 0.676 | 0.011 | 0 | Fibroblast | DCN |
| 8.231829 | 0.404 | 0.004 | 0 | Fibroblast | FBLN1 |
| 8.103953 | 0.4 | 0.004 | 0 | Fibroblast | FRZB |
| 7.986424 | 0.797 | 0.018 | 0 | Fibroblast | COL3A1 |
| 7.97707 | 0.569 | 0.009 | 0 | Fibroblast | SOD3 |
| 7.885567 | 0.8 | 0.019 | 0 | Fibroblast | COL1A2 |
| 7.882871 | 0.308 | 0.002 | 0 | Fibroblast | TBX2 |
| 7.874946 | 0.637 | 0.028 | 0 | Fibroblast | TAGLN |
| 7.856073 | 0.33 | 0.002 | 0 | Fibroblast | PRRX1 |
| 8.415646 | 0.351 | 0.004 | 0 | Endothelial | ACKR1 |
| 8.236392 | 0.499 | 0.002 | 0 | Endothelial | KDR |
| 8.210145 | 0.389 | 0.001 | 0 | Endothelial | TM4SF18 |
| 8.188199 | 0.663 | 0.003 | 0 | Endothelial | EMCN |
| 8.187329 | 0.558 | 0.003 | 0 | Endothelial | CLEC14A |
| 8.186499 | 0.507 | 0.003 | 0 | Endothelial | CLDN5 |
| 8.175677 | 0.54 | 0.002 | 0 | Endothelial | DIPK2B |
| 8.166976 | 0.275 | 0.001 | 0 | Endothelial | SOX17 |
| 8.165753 | 0.326 | 0.003 | 0 | Endothelial | CCL14 |
| 8.15329 | 0.37 | 0.002 | 0 | Endothelial | F2RL3 |
| 8.121868 | 0.32 | 0.001 | 0 | Endothelial | ARHGEF15 |
| 8.091594 | 0.606 | 0.002 | 0 | Endothelial | CDH5 |
| 8.067333 | 0.802 | 0.012 | 0 | Endothelial | PLVAP |
| 8.018397 | 0.755 | 0.009 | 0 | Endothelial | VWF |
| 7.994187 | 0.413 | 0.001 | 0 | Endothelial | MYCT1 |

**Table S5** Top10 marker genes of T/NK cell clusters.

| avg_log2FC | pct.1 | pct.2 | p_val_adj | cluster | gene |
| --- | --- | --- | --- | --- | --- |
| 1.942609 | 0.394 | 0.123 | 0 | CD4T_C0_IL7R | PLCB1 |
| 1.5106 | 0.831 | 0.466 | 0 | CD4T_C0_IL7R | IL7R |
| 1.499826 | 0.797 | 0.443 | 0 | CD4T_C0_IL7R | ANXA1 |
| 1.412119 | 0.25 | 0.094 | 0 | CD4T_C0_IL7R | CD40LG |
| 1.370138 | 0.442 | 0.179 | 0 | CD4T_C0_IL7R | ANK3 |
| 1.348181 | 0.259 | 0.116 | 0 | CD4T_C0_IL7R | MYBL1 |
| 1.333242 | 0.268 | 0.139 | 6.64E-243 | CD4T_C0_IL7R | CDKN1A |
| 1.329254 | 0.794 | 0.578 | 0 | CD4T_C0_IL7R | FOS |
| 1.279256 | 0.252 | 0.146 | 9.45E-154 | CD4T_C0_IL7R | CCL20 |
| 1.26557 | 0.446 | 0.265 | 0 | CD4T_C0_IL7R | LMNA |
| 1.233663 | 0.766 | 0.604 | 0 | CD4T_C0_IL7R | NFKBIA |
| 1.194854 | 0.819 | 0.645 | 0 | CD4T_C0_IL7R | JUN |
| 1.191709 | 0.626 | 0.443 | 0 | CD4T_C0_IL7R | FOSB |
| 1.102025 | 0.273 | 0.134 | 3.14E-275 | CD4T_C0_IL7R | SSBP2 |
| 1.09504 | 0.459 | 0.258 | 0 | CD4T_C0_IL7R | BACH2 |
| 6.571402 | 0.277 | 0.004 | 0 | NK_C0_TYROBP | KRT81 |
| 5.210099 | 0.614 | 0.022 | 0 | NK_C0_TYROBP | FCER1G |
| 4.267636 | 0.324 | 0.015 | 0 | NK_C0_TYROBP | SH2D1B |
| 4.15349 | 0.33 | 0.019 | 0 | NK_C0_TYROBP | KRT86 |
| 4.009154 | 0.685 | 0.052 | 0 | NK_C0_TYROBP | TYROBP |
| 3.854339 | 0.566 | 0.043 | 0 | NK_C0_TYROBP | TRDC |
| 3.619272 | 0.415 | 0.044 | 0 | NK_C0_TYROBP | KIR2DL4 |
| 3.584709 | 0.516 | 0.089 | 0 | NK_C0_TYROBP | XCL1 |
| 3.340564 | 0.357 | 0.042 | 0 | NK_C0_TYROBP | LAT2 |
| 3.108217 | 0.593 | 0.175 | 0 | NK_C0_TYROBP | AREG |
| 3.083364 | 0.294 | 0.042 | 0 | NK_C0_TYROBP | CD160 |
| 3.071221 | 0.277 | 0.034 | 0 | NK_C0_TYROBP | NCAM1 |
| 3.000643 | 0.565 | 0.144 | 0 | NK_C0_TYROBP | XCL2 |
| 2.929099 | 0.538 | 0.138 | 0 | NK_C0_TYROBP | GNLY |
| 2.70149 | 0.341 | 0.059 | 0 | NK_C0_TYROBP | KLRC3 |
| 2.702918 | 0.285 | 0.058 | 0 | CD8T_C4_PDCD1 | MYO1E |
| 2.63084 | 0.411 | 0.106 | 0 | CD8T_C4_PDCD1 | ATP8B4 |
| 2.520748 | 0.294 | 0.054 | 0 | CD8T_C4_PDCD1 | PDE7B |
| 2.423703 | 0.345 | 0.047 | 0 | CD8T_C4_PDCD1 | CXCL13 |
| 2.211334 | 0.308 | 0.07 | 0 | CD8T_C4_PDCD1 | CHN1 |
| 2.152354 | 0.481 | 0.138 | 0 | CD8T_C4_PDCD1 | ENTPD1 |
| 2.054418 | 0.356 | 0.108 | 0 | CD8T_C4_PDCD1 | DAPK2 |
| 1.973682 | 0.27 | 0.078 | 0 | CD8T_C4_PDCD1 | SRGAP3 |
| 1.942819 | 0.279 | 0.087 | 0 | CD8T_C4_PDCD1 | PLPP1 |
| 1.931463 | 0.292 | 0.125 | 0 | CD8T_C4_PDCD1 | NELL2 |
| 1.92743 | 0.302 | 0.099 | 0 | CD8T_C4_PDCD1 | TMIGD2 |
| 1.912355 | 0.339 | 0.109 | 0 | CD8T_C4_PDCD1 | TNFRSF9 |
| 1.909496 | 0.263 | 0.07 | 0 | CD8T_C4_PDCD1 | LAYN |
| 1.887414 | 0.718 | 0.311 | 0 | CD8T_C4_PDCD1 | LINC01871 |
| 1.808447 | 0.273 | 0.088 | 0 | CD8T_C4_PDCD1 | HAVCR2 |
| 3.832258 | 0.837 | 0.117 | 0 | CD8T_C3_GZMK | GZMK |
| 2.98545 | 0.253 | 0.048 | 0 | CD8T_C3_GZMK | IFNG-AS1 |
| 2.470419 | 0.338 | 0.091 | 0 | CD8T_C3_GZMK | KLRG1 |
| 2.348133 | 0.456 | 0.127 | 0 | CD8T_C3_GZMK | CRTAM |
| 2.230819 | 0.464 | 0.161 | 0 | CD8T_C3_GZMK | CMC1 |
| 2.103085 | 0.583 | 0.218 | 0 | CD8T_C3_GZMK | CCL4L2 |
| 1.895409 | 0.306 | 0.094 | 0 | CD8T_C3_GZMK | HLA-DQA1 |
| 1.848396 | 0.347 | 0.172 | 0 | CD8T_C3_GZMK | IFNG |
| 1.826897 | 0.4 | 0.151 | 0 | CD8T_C3_GZMK | SAMD3 |
| 1.77658 | 0.259 | 0.104 | 0 | CD8T_C3_GZMK | HLA-DRB5 |
| 1.714735 | 0.393 | 0.177 | 0 | CD8T_C3_GZMK | ITM2C |
| 1.696955 | 0.404 | 0.143 | 0 | CD8T_C3_GZMK | DTHD1 |
| 1.618948 | 0.476 | 0.197 | 0 | CD8T_C3_GZMK | GZMH |
| 1.592493 | 0.797 | 0.448 | 0 | CD8T_C3_GZMK | CST7 |
| 1.578952 | 0.495 | 0.218 | 0 | CD8T_C3_GZMK | HLA-DRB1 |
| 5.628433 | 0.428 | 0.013 | 0 | CD4T_C2_FOXP3 | FOXP3 |
| 5.53571 | 0.282 | 0.009 | 0 | CD4T_C2_FOXP3 | IL1R2 |
| 4.242975 | 0.369 | 0.027 | 0 | CD4T_C2_FOXP3 | IL1R1 |
| 4.020167 | 0.256 | 0.024 | 0 | CD4T_C2_FOXP3 | RTKN2 |
| 3.830015 | 0.463 | 0.064 | 0 | CD4T_C2_FOXP3 | IL2RA |
| 3.624777 | 0.472 | 0.084 | 0 | CD4T_C2_FOXP3 | TNFRSF4 |
| 3.248313 | 0.334 | 0.056 | 0 | CD4T_C2_FOXP3 | LINC01943 |
| 2.861652 | 0.605 | 0.138 | 0 | CD4T_C2_FOXP3 | TBC1D4 |
| 2.784755 | 0.495 | 0.123 | 0 | CD4T_C2_FOXP3 | TNFRSF18 |
| 2.730054 | 0.363 | 0.125 | 0 | CD4T_C2_FOXP3 | CRADD |
| 2.728987 | 0.786 | 0.29 | 0 | CD4T_C2_FOXP3 | BATF |
| 2.694695 | 0.423 | 0.079 | 0 | CD4T_C2_FOXP3 | GK |
| 2.679415 | 0.363 | 0.072 | 0 | CD4T_C2_FOXP3 | ICA1 |
| 2.672265 | 0.287 | 0.053 | 0 | CD4T_C2_FOXP3 | LAIR2 |
| 2.608227 | 0.485 | 0.123 | 0 | CD4T_C2_FOXP3 | LINC02694 |
| 5.104306 | 0.587 | 0.023 | 0 | CD8T_C6_SLC4A10 | SLC4A10 |
| 4.636438 | 0.322 | 0.015 | 0 | CD8T_C6_SLC4A10 | TRAV1-2 |
| 3.392393 | 0.406 | 0.051 | 0 | CD8T_C6_SLC4A10 | NCR3 |
| 2.940756 | 0.4 | 0.059 | 0 | CD8T_C6_SLC4A10 | IL4I1 |
| 2.895348 | 0.289 | 0.041 | 0 | CD8T_C6_SLC4A10 | TLE1 |
| 2.790368 | 0.573 | 0.099 | 0 | CD8T_C6_SLC4A10 | ZBTB16 |
| 2.717144 | 0.257 | 0.037 | 0 | CD8T_C6_SLC4A10 | LST1 |
| 2.695389 | 0.478 | 0.15 | 0 | CD8T_C6_SLC4A10 | CCL20 |
| 2.631576 | 0.448 | 0.102 | 0 | CD8T_C6_SLC4A10 | CEBPD |
| 2.179008 | 0.376 | 0.091 | 0 | CD8T_C6_SLC4A10 | HPGD |
| 2.168529 | 0.256 | 0.052 | 0 | CD8T_C6_SLC4A10 | DPP4 |
| 2.15783 | 0.292 | 0.059 | 0 | CD8T_C6_SLC4A10 | IL23R |
| 1.92308 | 0.427 | 0.111 | 0 | CD8T_C6_SLC4A10 | TMIGD2 |
| 1.878684 | 0.932 | 0.442 | 0 | CD8T_C6_SLC4A10 | KLRB1 |
| 1.641989 | 0.387 | 0.132 | 4.54E-237 | CD8T_C6_SLC4A10 | SPRY1 |
| 3.144374 | 0.283 | 0.053 | 0 | CD4T_C3_TOX2 | PTPN13 |
| 2.780614 | 0.461 | 0.094 | 0 | CD4T_C3_TOX2 | TSHZ2 |
| 2.442243 | 0.363 | 0.096 | 0 | CD4T_C3_TOX2 | TOX2 |
| 2.321119 | 0.359 | 0.11 | 0 | CD4T_C3_TOX2 | SESN3 |
| 2.304787 | 0.448 | 0.159 | 0 | CD4T_C3_TOX2 | SLC9A9 |
| 2.291957 | 0.391 | 0.12 | 0 | CD4T_C3_TOX2 | FAAH2 |
| 2.218625 | 0.297 | 0.083 | 0 | CD4T_C3_TOX2 | LRRC8D |
| 2.208559 | 0.367 | 0.097 | 0 | CD4T_C3_TOX2 | ICA1 |
| 2.12237 | 0.401 | 0.14 | 0 | CD4T_C3_TOX2 | TNFSF8 |
| 2.08845 | 0.361 | 0.114 | 0 | CD4T_C3_TOX2 | GK |
| 1.880877 | 0.252 | 0.086 | 0 | CD4T_C3_TOX2 | ABTB3 |
| 1.867057 | 0.305 | 0.1 | 0 | CD4T_C3_TOX2 | CD40LG |
| 1.780386 | 0.378 | 0.181 | 2.16E-300 | CD4T_C3_TOX2 | THADA |
| 1.759571 | 0.275 | 0.094 | 0 | CD4T_C3_TOX2 | TIAM1 |
| 1.687486 | 0.54 | 0.283 | 0 | CD4T_C3_TOX2 | IL6ST |
| 3.272298 | 0.318 | 0.04 | 0 | CD8T_C5_TRDC | CD160 |
| 3.246801 | 0.386 | 0.043 | 0 | CD8T_C5_TRDC | KIR2DL4 |
| 3.088799 | 0.603 | 0.09 | 0 | CD8T_C5_TRDC | KLRC2 |
| 3.035728 | 0.421 | 0.054 | 0 | CD8T_C5_TRDC | KLRC3 |
| 2.861801 | 0.493 | 0.095 | 0 | CD8T_C5_TRDC | LINC02446 |
| 2.802711 | 0.339 | 0.051 | 0 | CD8T_C5_TRDC | TRDC |
| 2.540773 | 0.35 | 0.067 | 0 | CD8T_C5_TRDC | PELO-AS1 |
| 2.173561 | 0.291 | 0.066 | 0 | CD8T_C5_TRDC | PIK3AP1 |
| 2.047695 | 0.771 | 0.191 | 0 | CD8T_C5_TRDC | KLRD1 |
| 2.030878 | 0.633 | 0.19 | 0 | CD8T_C5_TRDC | HOPX |
| 2.029036 | 0.363 | 0.104 | 0 | CD8T_C5_TRDC | KLRC1 |
| 1.992993 | 0.318 | 0.095 | 0 | CD8T_C5_TRDC | XCL1 |
| 1.975345 | 0.395 | 0.143 | 0 | CD8T_C5_TRDC | GNLY |
| 1.961183 | 0.364 | 0.091 | 0 | CD8T_C5_TRDC | CHN2 |
| 1.933319 | 0.315 | 0.077 | 0 | CD8T_C5_TRDC | PLCG2 |
| 2.831854 | 0.316 | 0.049 | 0 | CD8T_C2_ZNF683 | ZNF683 |
| 2.040565 | 0.383 | 0.088 | 0 | CD8T_C2_ZNF683 | KLRC1 |
| 2.037671 | 0.346 | 0.088 | 0 | CD8T_C2_ZNF683 | LINC02446 |
| 1.936225 | 0.672 | 0.193 | 0 | CD8T_C2_ZNF683 | CD8B |
| 1.851942 | 0.766 | 0.264 | 0 | CD8T_C2_ZNF683 | CD8A |
| 1.590626 | 0.275 | 0.087 | 0 | CD8T_C2_ZNF683 | XCL1 |
| 1.569714 | 0.526 | 0.234 | 0 | CD8T_C2_ZNF683 | CCL4L2 |
| 1.567899 | 0.73 | 0.257 | 0 | CD8T_C2_ZNF683 | GZMB |
| 1.551288 | 0.401 | 0.135 | 0 | CD8T_C2_ZNF683 | XCL2 |
| 1.48189 | 0.506 | 0.178 | 0 | CD8T_C2_ZNF683 | HOPX |
| 1.479055 | 0.499 | 0.182 | 0 | CD8T_C2_ZNF683 | ITGA1 |
| 1.421379 | 0.453 | 0.193 | 0 | CD8T_C2_ZNF683 | CAPG |
| 1.418303 | 0.273 | 0.134 | 5.81E-214 | CD8T_C2_ZNF683 | CCL3L3 |
| 1.385633 | 0.836 | 0.394 | 0 | CD8T_C2_ZNF683 | CCL4 |
| 1.315941 | 0.273 | 0.125 | 1.11E-257 | CD8T_C2_ZNF683 | SPRY1 |
| 8.060463 | 0.622 | 0.006 | 0 | NK_C1_FGFBP2 | FGFBP2 |
| 6.654762 | 0.597 | 0.013 | 0 | NK_C1_FGFBP2 | FCGR3A |
| 5.768533 | 0.27 | 0.006 | 0 | NK_C1_FGFBP2 | PRSS23 |
| 5.645103 | 0.373 | 0.011 | 0 | NK_C1_FGFBP2 | S1PR5 |
| 5.283391 | 0.458 | 0.015 | 0 | NK_C1_FGFBP2 | KLRF1 |
| 3.847138 | 0.504 | 0.046 | 0 | NK_C1_FGFBP2 | C1orf21 |
| 3.778113 | 0.563 | 0.051 | 0 | NK_C1_FGFBP2 | TYROBP |
| 3.563932 | 0.264 | 0.034 | 0 | NK_C1_FGFBP2 | SLC15A4 |
| 3.439236 | 0.843 | 0.12 | 0 | NK_C1_FGFBP2 | GNLY |
| 3.281548 | 0.315 | 0.044 | 0 | NK_C1_FGFBP2 | ADGRG1 |
| 3.163197 | 0.423 | 0.061 | 0 | NK_C1_FGFBP2 | PLEK |
| 3.105122 | 0.448 | 0.12 | 0 | NK_C1_FGFBP2 | CCL3 |
| 3.025974 | 0.273 | 0.041 | 0 | NK_C1_FGFBP2 | LYN |
| 2.971162 | 0.441 | 0.125 | 0 | NK_C1_FGFBP2 | SPON2 |
| 2.955658 | 0.811 | 0.256 | 0 | NK_C1_FGFBP2 | PRF1 |
| 3.219279 | 0.43 | 0.061 | 0 | CD4T_C1_SELL | LEF1 |
| 2.486705 | 0.514 | 0.116 | 0 | CD4T_C1_SELL | CCR7 |
| 2.314038 | 0.364 | 0.099 | 0 | CD4T_C1_SELL | SELL |
| 2.098296 | 0.376 | 0.092 | 0 | CD4T_C1_SELL | TSHZ2 |
| 1.862625 | 0.315 | 0.089 | 0 | CD4T_C1_SELL | PLAC8 |
| 1.741207 | 0.456 | 0.194 | 0 | CD4T_C1_SELL | KLF2 |
| 1.718453 | 0.402 | 0.143 | 0 | CD4T_C1_SELL | TCF7 |
| 1.61933 | 0.331 | 0.117 | 0 | CD4T_C1_SELL | FAAH2 |
| 1.618305 | 0.314 | 0.143 | 0 | CD4T_C1_SELL | ARHGEF18 |
| 1.57726 | 0.324 | 0.117 | 0 | CD4T_C1_SELL | TXK |
| 1.55075 | 0.259 | 0.112 | 7.61E-294 | CD4T_C1_SELL | SESN3 |
| 1.486014 | 0.27 | 0.119 | 1.37E-298 | CD4T_C1_SELL | AP3M2 |
| 1.469445 | 0.306 | 0.15 | 7.84E-279 | CD4T_C1_SELL | PRKCQ-AS1 |
| 1.432243 | 0.451 | 0.186 | 0 | CD4T_C1_SELL | ANK3 |
| 1.424952 | 0.362 | 0.191 | 0 | CD4T_C1_SELL | NOSIP |
| 7.66178 | 0.52 | 0.004 | 0 | CD8T_C1_MKI67 | UBE2C |
| 7.533892 | 0.33 | 0.002 | 0 | CD8T_C1_MKI67 | DLGAP5 |
| 7.480798 | 0.303 | 0.001 | 0 | CD8T_C1_MKI67 | CDCA3 |
| 7.371069 | 0.317 | 0.004 | 0 | CD8T_C1_MKI67 | H3C2 |
| 7.368729 | 0.308 | 0.002 | 0 | CD8T_C1_MKI67 | CDC20 |
| 7.368015 | 0.402 | 0.002 | 0 | CD8T_C1_MKI67 | AURKB |
| 7.225888 | 0.39 | 0.002 | 0 | CD8T_C1_MKI67 | GTSE1 |
| 7.165031 | 0.321 | 0.002 | 0 | CD8T_C1_MKI67 | SPC25 |
| 7.040371 | 0.531 | 0.007 | 0 | CD8T_C1_MKI67 | RRM2 |
| 6.898836 | 0.35 | 0.003 | 0 | CD8T_C1_MKI67 | CCNB2 |
| 6.83621 | 0.469 | 0.003 | 0 | CD8T_C1_MKI67 | BIRC5 |
| 6.828087 | 0.283 | 0.002 | 0 | CD8T_C1_MKI67 | PKMYT1 |
| 6.620936 | 0.379 | 0.012 | 0 | CD8T_C1_MKI67 | H1-5 |
| 6.437539 | 0.278 | 0.002 | 0 | CD8T_C1_MKI67 | SHCBP1 |
| 6.417142 | 0.418 | 0.007 | 0 | CD8T_C1_MKI67 | ASPM |

**Table S6** Top10 marker genes of B cell clusters.

| avg_log2FC | pct.1 | pct.2 | p_val_adj | cluster | gene |
| --- | --- | --- | --- | --- | --- |
| 2.835909 | 0.422 | 0.07 | 0 | B_C0_TCL1A | TCL1A |
| 2.835401 | 0.449 | 0.086 | 0 | B_C0_TCL1A | IGHD |
| 2.469925 | 0.312 | 0.088 | 0 | B_C0_TCL1A | IL4R |
| 2.362613 | 0.364 | 0.102 | 0 | B_C0_TCL1A | FCER2 |
| 1.99122 | 0.308 | 0.113 | 0 | B_C0_TCL1A | COL19A1 |
| 1.833274 | 0.25 | 0.101 | 0 | B_C0_TCL1A | CLEC2B |
| 1.696811 | 0.37 | 0.159 | 0 | B_C0_TCL1A | CAMK2D |
| 1.50897 | 0.272 | 0.126 | 4.20E-298 | B_C0_TCL1A | MTSS1 |
| 1.385444 | 0.263 | 0.144 | 5.36E-206 | B_C0_TCL1A | MARCHF3 |
| 1.366601 | 0.288 | 0.164 | 4.04E-212 | B_C0_TCL1A | LARGE1 |
| 1.309831 | 0.352 | 0.216 | 2.44E-243 | B_C0_TCL1A | TMEM123 |
| 1.293099 | 0.377 | 0.223 | 1.68E-291 | B_C0_TCL1A | CLEC2D |
| 1.289087 | 0.381 | 0.251 | 3.46E-243 | B_C0_TCL1A | FCRL1 |
| 1.265597 | 0.403 | 0.23 | 0 | B_C0_TCL1A | PHACTR1 |
| 1.205636 | 0.41 | 0.278 | 1.34E-242 | B_C0_TCL1A | PRKCE |
| 1.426815 | 0.307 | 0.125 | 0 | B_C2_SNED1 | SNED1 |
| 1.379615 | 0.301 | 0.151 | 5.39E-233 | B_C2_SNED1 | LNCRNA-IUR |
| 1.373546 | 0.263 | 0.104 | 0 | B_C2_SNED1 | GAB2 |
| 1.355093 | 0.569 | 0.252 | 0 | B_C2_SNED1 | SAMSN1 |
| 1.318706 | 0.412 | 0.207 | 0 | B_C2_SNED1 | SESN3 |
| 1.280238 | 0.466 | 0.211 | 0 | B_C2_SNED1 | RGS2 |
| 1.214902 | 0.357 | 0.143 | 0 | B_C2_SNED1 | TNFRSF13B |
| 1.178411 | 0.251 | 0.108 | 1.96E-247 | B_C2_SNED1 | NR4A3 |
| 1.131622 | 0.724 | 0.385 | 0 | B_C2_SNED1 | GPR183 |
| 1.02082 | 0.26 | 0.126 | 3.63E-197 | B_C2_SNED1 | LINC01857 |
| 2.338086 | 0.607 | 0.17 | 0 | B_C4_HSPA1A | HSPA1A |
| 2.301182 | 0.544 | 0.162 | 0 | B_C4_HSPA1A | HSPA1B |
| 1.88777 | 0.268 | 0.147 | 1.80E-101 | B_C4_HSPA1A | HSPB1 |
| 1.861327 | 0.684 | 0.325 | 0 | B_C4_HSPA1A | DNAJB1 |
| 1.664831 | 0.325 | 0.173 | 4.13E-150 | B_C4_HSPA1A | HSPH1 |
| 1.633948 | 0.65 | 0.391 | 0 | B_C4_HSPA1A | HSPE1 |
| 1.609489 | 0.354 | 0.161 | 7.89E-220 | B_C4_HSPA1A | LINC01781 |
| 1.388328 | 0.837 | 0.638 | 0 | B_C4_HSPA1A | HSP90AA1 |
| 1.234816 | 0.4 | 0.278 | 2.56E-96 | B_C4_HSPA1A | HSPD1 |
| 1.117459 | 0.412 | 0.279 | 2.01E-98 | B_C4_HSPA1A | OSBPL10 |
| 1.033586 | 0.261 | 0.183 | 5.80E-41 | B_C4_HSPA1A | DENND5B |
| 3.664285 | 0.359 | 0.068 | 0 | B_C5_EGR1 | EGR1 |
| 3.570147 | 0.48 | 0.084 | 0 | B_C5_EGR1 | TEX14 |
| 2.827596 | 0.411 | 0.088 | 0 | B_C5_EGR1 | LINC00910 |
| 2.625992 | 0.454 | 0.171 | 0 | B_C5_EGR1 | DUSP2 |
| 2.559636 | 0.259 | 0.06 | 0 | B_C5_EGR1 | TMEM107 |
| 2.275493 | 0.616 | 0.283 | 0 | B_C5_EGR1 | NFKBIA |
| 2.120512 | 0.253 | 0.085 | 1.03E-242 | B_C5_EGR1 | SERTAD1 |
| 2.054016 | 0.279 | 0.102 | 1.99E-239 | B_C5_EGR1 | EIF4A3 |
| 2.000007 | 0.67 | 0.347 | 0 | B_C5_EGR1 | IER2 |
| 1.814739 | 0.653 | 0.35 | 0 | B_C5_EGR1 | FOSB |
| 1.678844 | 0.288 | 0.129 | 2.43E-161 | B_C5_EGR1 | MIR155HG |
| 1.631768 | 0.36 | 0.179 | 6.60E-178 | B_C5_EGR1 | GADD45B |
| 1.579847 | 0.695 | 0.431 | 0 | B_C5_EGR1 | ZFP36 |
| 1.566824 | 0.688 | 0.35 | 0 | B_C5_EGR1 | NR4A1 |
| 1.542063 | 0.496 | 0.254 | 3.75E-273 | B_C5_EGR1 | BCL2A1 |
| 2.676781 | 0.386 | 0.082 | 0 | B_C3_KYNU | HIPK2 |
| 2.417829 | 0.628 | 0.179 | 0 | B_C3_KYNU | PDE4D |
| 2.415408 | 0.282 | 0.059 | 0 | B_C3_KYNU | SSPN |
| 2.28681 | 0.267 | 0.06 | 0 | B_C3_KYNU | TEX9 |
| 2.198461 | 0.289 | 0.062 | 0 | B_C3_KYNU | TOX |
| 1.956058 | 0.426 | 0.138 | 0 | B_C3_KYNU | KYNU |
| 1.500545 | 0.708 | 0.351 | 0 | B_C3_KYNU | CRIP1 |
| 1.342675 | 0.252 | 0.104 | 3.32E-169 | B_C3_KYNU | AHNAK |
| 1.313204 | 0.293 | 0.12 | 1.53E-203 | B_C3_KYNU | SCIMP |
| 1.182967 | 0.286 | 0.127 | 6.21E-166 | B_C3_KYNU | ANK3 |
| 1.165647 | 0.259 | 0.117 | 3.12E-142 | B_C3_KYNU | ZDHHC14 |
| 1.147477 | 0.434 | 0.229 | 3.66E-200 | B_C3_KYNU | VIM |
| 1.126178 | 0.306 | 0.138 | 1.71E-172 | B_C3_KYNU | S100A10 |
| 1.107144 | 0.395 | 0.198 | 2.00E-192 | B_C3_KYNU | CD99 |
| 1.106976 | 0.426 | 0.208 | 1.30E-218 | B_C3_KYNU | CYRIA |
| 5.73514 | 0.305 | 0.012 | 1.05E-225 | B_C1_GKN2 | GKN2 |
| 5.69002 | 0.338 | 0.02 | 9.15E-160 | B_C1_GKN2 | GKN1 |
| 4.574863 | 0.571 | 0.095 | 2.50E-95 | B_C1_GKN2 | TFF1 |
| 4.405737 | 0.435 | 0.034 | 7.15E-159 | B_C1_GKN2 | IFI27 |
| 4.369448 | 0.279 | 0.024 | 3.45E-87 | B_C1_GKN2 | LCN2 |
| 4.303425 | 0.383 | 0.041 | 5.96E-97 | B_C1_GKN2 | TFF2 |
| 4.265614 | 0.253 | 0.027 | 2.74E-62 | B_C1_GKN2 | IFITM3 |
| 3.742005 | 0.494 | 0.073 | 7.39E-90 | B_C1_GKN2 | AGR2 |
| 3.73162 | 0.318 | 0.037 | 2.33E-72 | B_C1_GKN2 | KRT8 |
| 3.728973 | 0.279 | 0.037 | 1.05E-52 | B_C1_GKN2 | SPINK1 |
| 3.627805 | 0.292 | 0.029 | 6.63E-79 | B_C1_GKN2 | CST3 |
| 3.4909 | 0.409 | 0.131 | 6.54E-23 | B_C1_GKN2 | PGC |
| 2.824704 | 0.468 | 0.111 | 5.21E-44 | B_C1_GKN2 | LYZ |
| 2.341139 | 0.558 | 0.272 | 1.91E-19 | B_C1_GKN2 | S100A6 |
| 1.96043 | 0.253 | 0.103 | 3.00E-06 | B_C1_GKN2 | S100A11 |
| 1.140831 | 0.259 | 0.146 | 1.72E-10 | B_C8_S100A4 | S100A4 |
| 7.829574 | 0.58 | 0.004 | 0 | B_C7_RGS13 | CCDC144BP |
| 7.530367 | 0.37 | 0.002 | 0 | B_C7_RGS13 | MYBL1 |
| 7.122974 | 0.313 | 0.002 | 0 | B_C7_RGS13 | DCAF12 |
| 7.018247 | 0.746 | 0.012 | 0 | B_C7_RGS13 | RGS13 |
| 6.609643 | 0.279 | 0.002 | 0 | B_C7_RGS13 | SLCO3A1 |
| 6.47169 | 0.313 | 0.003 | 0 | B_C7_RGS13 | NUGGC |
| 6.403197 | 0.649 | 0.01 | 0 | B_C7_RGS13 | MEF2B |
| 6.19108 | 0.448 | 0.006 | 0 | B_C7_RGS13 | SERPINA9 |
| 6.184651 | 0.544 | 0.01 | 0 | B_C7_RGS13 | RAPGEF5 |
| 6.076834 | 0.304 | 0.004 | 0 | B_C7_RGS13 | CDCA7 |
| 6.053624 | 0.394 | 0.007 | 0 | B_C7_RGS13 | EPS15-AS1 |
| 6.028991 | 0.276 | 0.003 | 0 | B_C7_RGS13 | ACY3 |
| 5.973254 | 0.307 | 0.005 | 0 | B_C7_RGS13 | PCLAF |
| 5.79415 | 0.366 | 0.007 | 0 | B_C7_RGS13 | SUGCT |
| 5.643986 | 0.269 | 0.005 | 0 | B_C7_RGS13 | LMO2 |
| 9.695303 | 0.847 | 0.011 | 0 | B_C6_HBA1 | HBA1 |
| 9.60781 | 0.88 | 0.027 | 0 | B_C6_HBA1 | HBA2 |
| 8.890992 | 0.894 | 0.089 | 0 | B_C6_HBA1 | HBB |
